# Multimodal single-cell analyses reveal distinct fusion-regulated transcriptional programs in Ewing sarcoma

**DOI:** 10.7554/eLife.109124

**Published:** 2026-11-11

**Authors:** Olivia G Waltner, April A Apfelbaum, Emma D Wrenn, Shruti S Bhise, Sami B Kanaan, Rula Green Gladden, Mark A Mendoza, Roger Volden, Zev Kronenberg, Anand G Patel, Michael A Dyer, Jay F Sarthy, Elizabeth R Lawlor, Scott N Furlan

**Affiliations:** 1 Translational Science and Therapeutics Division, Fred Hutch Cancer Center Seattle United States; 2 Ben Towne Center for Childhood Cancer and Blood Disorders Research, Seattle Children’s Research Institute Seattle United States; 3 Pacific Biosciences Menlo Park United States; 4 St. Jude Children's Research Hospital Memphis United States; 5 Department of Pediatrics, University of Washington Seattle United States; Quest University Canada Canada; Quest University Canada Canada

**Keywords:** Ewing sarcoma, tumor cell plasticity, TGF-β signaling, gene-regulatory networks, single-cell multiomics, Human

## Abstract

Ewing sarcoma (EwS) is a fusion-driven malignancy, peaking in adolescence. Although EwS tumors are driven uniquely by EWS::FLI1 and related fusions, patient outcomes vary greatly. If and how tumor plasticity of EWS::FLI1-regulated transcriptional signatures contributes to disease progression is not known. To address this, we utilized a single-cell co-assay of RNA and chromatin accessibility (ATAC) sequencing to identify gene regulatory networks in EwS. By comprehensively characterizing regulatory elements across cell lines, we identified multiple unique modules of gene regulation. Differential usage and prevalence of these modules was evident across cell lines, associated with distinct epigenetic and transcriptomic signatures, and in specific cases, modifiable by exogenous TGF-β. When we examined primary EwS patient tumors, we observed these same regulatory modules were variably enriched both across and within tumors, highlighting the existence of intratumoral heterogeneity in gene regulatory networks. Our findings demonstrate that multiple, co-existing transcriptional programs shape the phenotypic diversity of EwS and suggest that the balance between these networks may have important implications for clinical outcomes and targeted therapy development.

## Introduction

Ewing sarcoma (EwS) is an aggressive, fusion-driven bone and soft tissue tumor encountered most in adolescents and young adults. Over a third of patients, including nearly all with metastatic or recurrent disease, do not survive (Riggi et al., 2021; Grünewald et al., 2018). Clinical presentation of EwS is heterogeneous, with 80% of localized cases presenting in the bone, and 20% arising in extraosseous locations. Histologically, EwS tumors are highly undifferentiated, exhibiting phenotypic features of both mesenchymal and neural crest progenitors. Although the cell of origin is controversial, EwS tumors are presumed to be derived from these lineages (Tirode et al., 2007; Riggi et al., 2009; Lin et al., 2011; Miller et al., 2020). Chromosomal translocations that create fusions between the N-terminal domain of *EWSR1* and the DNA-binding domain of ETS transcription factor (TF) family members are the primary oncogenic lesion in EwS (Riggi et al., 2021). The resulting EWS::ETS chimeric oncoprotein reprograms gene regulatory networks through diverse mechanisms, including modifying chromatin state through its function as a pioneer factor at GGAA microsatellite repeats, creating de novo enhancers (Riggi et al., 2014; Boulay et al., 2017). Apart from their fusions, EwS tumors are relatively bereft of mutations (Riggi et al., 2021; Crompton et al., 2014). Neither additional genetic alterations, nor differences in fusion breakpoint and binding partner heterogeneity (Zoubek et al., 1996) adequately explain the diverse clinical outcomes that are observed in patients who receive identical standard-of-care therapies. Advances in transcriptomic and epigenomic analyses have in recent years demonstrated the profound contributions of tumor cell plasticity and cell state heterogeneity to tumor metastasis, relapse, and therapy resistance (Davies et al., 2023; Hanahan, 2022). EwS tumors demonstrate marked inter- and intra-tumoral heterogeneity in their epigenetic states (Tomazou et al., 2015; Sheffield et al., 2017) and, as such, these differences may be key determinants of disease progression and clinical outcome.

Recent studies by our group and others have used single-cell genomics to study cellular heterogeneity within EwS tumors (Sheffield et al., 2017; Franzetti et al., 2017; Adane et al., 2021; Pedersen et al., 2016; Surdez et al., 2021; Aynaud et al., 2020; Apfelbaum et al., 2022b; Wrenn et al., 2023; Visser et al., 2023; Goodspeed et al., 2025). These studies reveal that EwS tumors coopt distinct developmental programs to promote proliferation or migratory behavior (Franzetti et al., 2017; Adane et al., 2021; Surdez et al., 2021; Apfelbaum et al., 2022b; Wrenn et al., 2023; Chaturvedi et al., 2012; Chaturvedi et al., 2014). In addition, accruing evidence shows that cooperativity between TFs, EWS::FLI1, and diverse chromatin states may underlie these various oncogenic programs (Apfelbaum et al., 2022b; Smith et al., 2006; Bilke et al., 2013; Bledsoe et al., 2014; García-García et al., 2021; Lin et al., 2019; Marques Howarth et al., 2014; Shi et al., 2020; Kinsey et al., 2006; Sanalkumar et al., 2023). We hypothesized that joint single-cell transcriptional and chromatin profiling technology would reveal new insight into the cell-intrinsic epigenetic and transcriptional heterogeneity of EwS cell states. Using this approach, we have identified a set of diverse gene regulatory networks that are differentially influenced by the EWS::FLI1 fusion and TGF-β in the tumor microenvironment. These distinctive networks vary both across and within tumors from EwS patients, providing evidence that a diversity of gene regulatory programs may contribute to the heterogeneity of this disease.

## Results

### Multiomic analyses reveal distinct chromatin accessibility states across EwS cell lines

**Figure 1.**
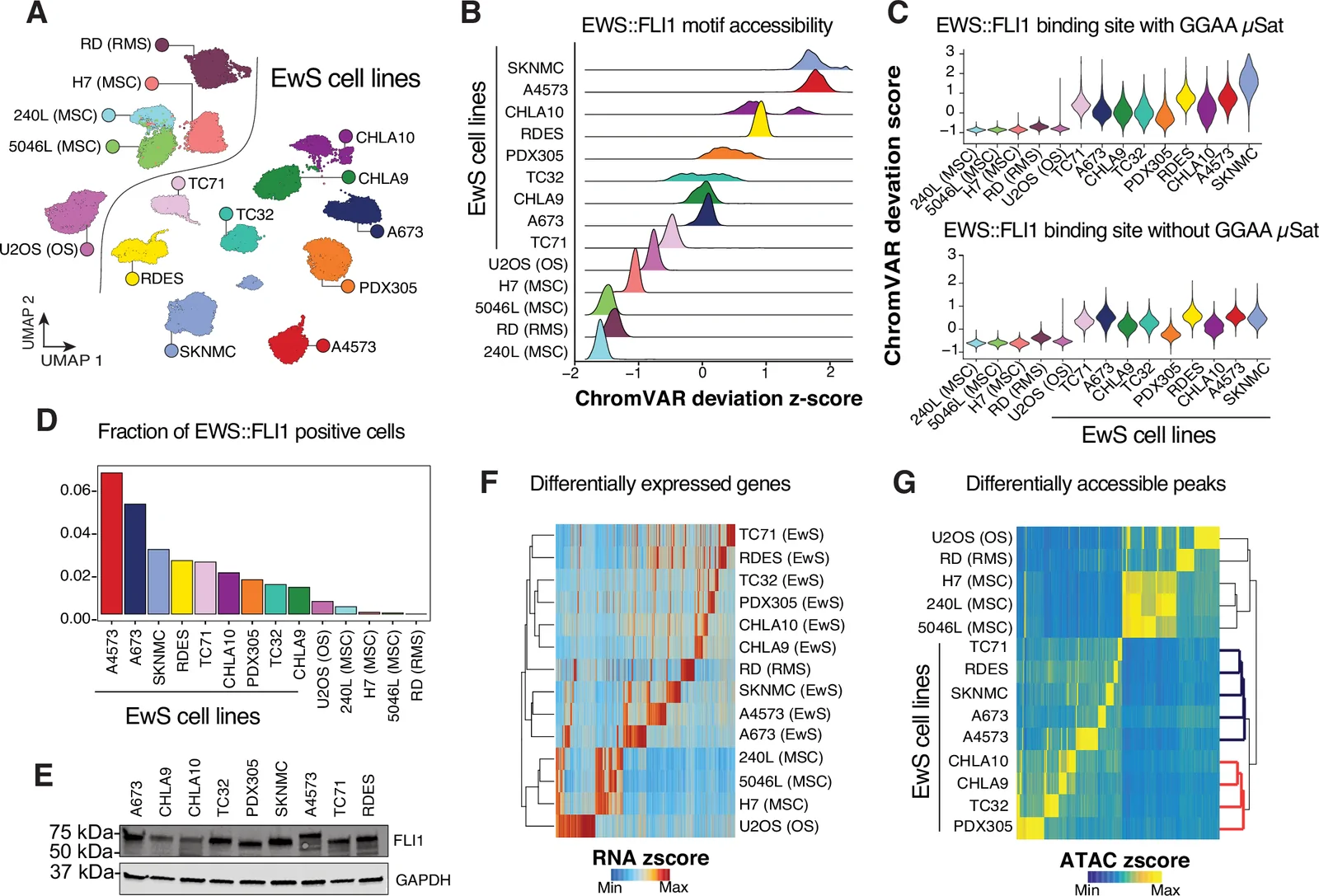
Multiomic analyses reveal distinct signatures between non-EwS and EwS cells. (**A**) UMAP embedding of single-cell RNA sequencing and ATAC sequencing of EwS and non-EwS cells. (**B**) ChromVAR deviation scores of EWS::FLI1 motif enrichment across EwS and non-EwS cells. (**C**) ChromVAR deviation scores of EWS::FLI1 binding site enrichment, separated by regions with or without GGAA microsatellites (µSAT; GGAA repeat ≥4), grouped by cell line. (**D**) Fraction of cells in each cell line with detection of EWS::FLI1 fusion transcript. (**E**) Western blot of FLI1 across EwS cell lines. GAPDH = loading control. (**F**) Differentially expressed genes (FDR ≤0.01 and log_2_FC ≥1.25) across EwS/non-EwS landscape. (**G**) Differentially accessible peaks (FDR ≤0.01 and log_2_FC ≥1.25) of EwS and non-EwS cells with row dendrogram showing phenotypic clusters. Row dendrogram shows EwS cell clusters highlighted in bold and red. Figure 1—source data 1.PDF file containing original western blots for Figure 1E, indicating the relevant bands and cell lines. Figure 1—source data 2.Original files for western blot analysis displayed in Figure 1E (FLI1 and GAPDH).

Cell lines are one of the few available models used to interrogate EwS biology given the rarity of the disease and the lack of appropriately faithful animal models (Minas et al., 2017). One drawback of cell line use is potential loss of intra-tumoral heterogeneity compared to the original tumor due to serial passaging. We reasoned that profiling an array of EwS cell lines would capture the diversity of gene regulatory networks that may underlie the clinical and transcriptional heterogeneity of this disease. Because of the well-described direct influence of the EwS fusion on chromatin remodeling (Sanalkumar et al., 2023), we utilized an assay capable of pairing single-cell RNA transcript counts with a chromatin assay for transposase-accessible chromatin (ATAC) (herein referred to as ‘multiomic’ sequencing) on nine EwS and five non-EwS cell lines (Figure 1A, Figure 1—figure supplement 1A). We recovered 30,105 profiles after filtering for poor quality cells (Figure 1A, Figure 1—figure supplement 1A, B). Multiomic single-cell profiles self-assembled after dimensionality reduction according to cell line identity (Figure 1A).

**Figure 1—figure supplement 1.**
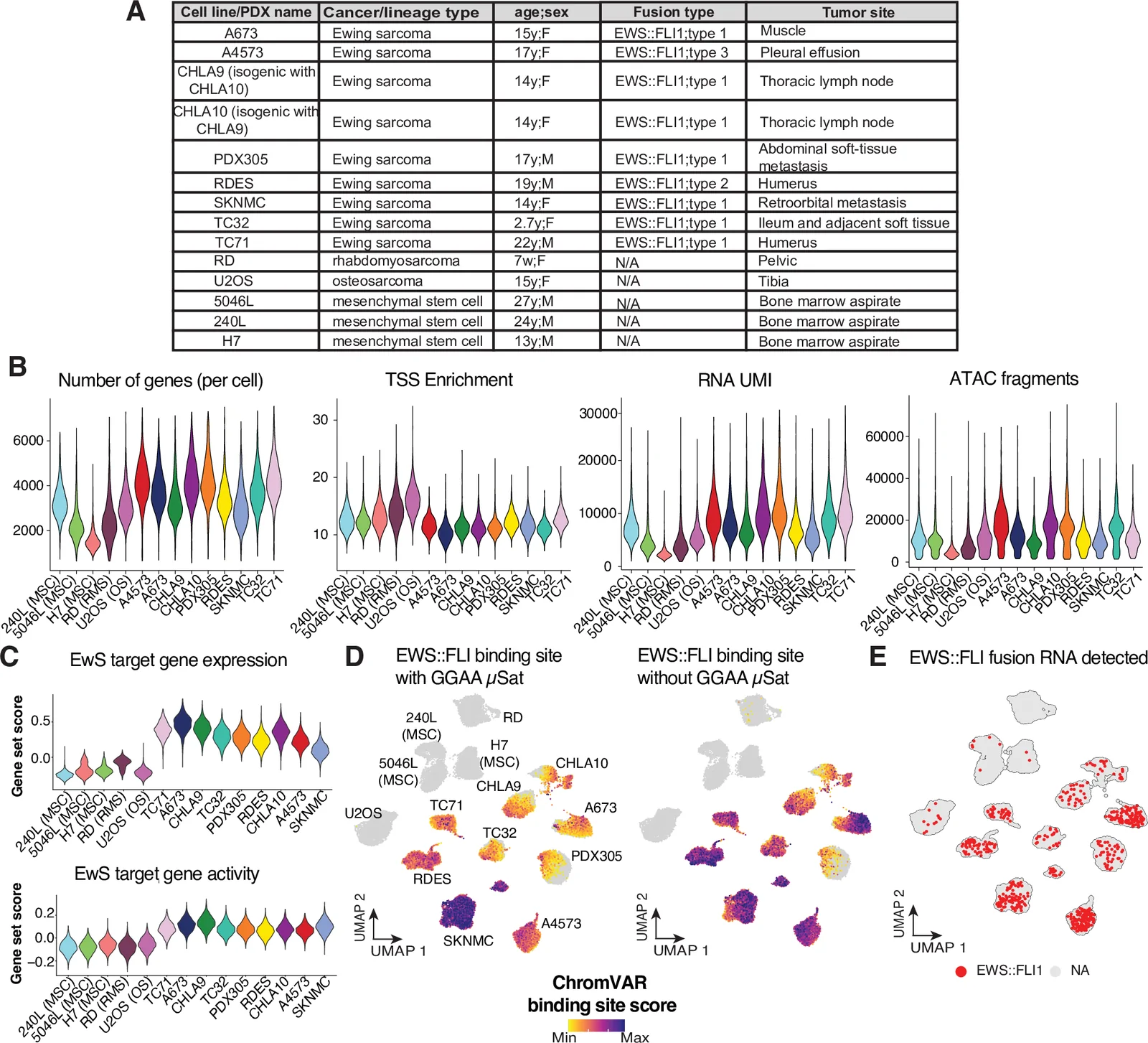
Multiomic analyses reveal distinct signatures between non-EwS and EwS cells. (**A**) Table of all 14 cell lines used for the multiome sequencing. (**B**) Quality control metrics across cell lines. (**C**) Gene set expression and activity score of EwS target genes. (**D**) UMAP of all Ewing and non-Ewing cell lines colored by chromVAR deviation scores of EWS::FLI1 binding sites, parsed by regions with or without GGAA microsatellites (µSAT; GGAA repeat ≥4). (**E**) UMAP of EwS and non-EwS cells highlighted (red) if EWS::FLI1 fusion transcript was detected by long-read sequencing.

First, to identify the extent of transcriptional heterogeneity of validated direct EWS::FLI1 target genes (EwS TGs; Aynaud et al., 2020) in individual cells, we calculated expression of EwS TGs at the single-cell level, subtracted by the aggregated expression of control gene sets (Tirosh et al., 2016). As expected, RNA expression of these EwS TGs was higher in EwS compared to non-EwS cell lines (Figure 1—figure supplement 1C). As these published EwS TGs were experimentally derived from the A673 cell line (Aynaud et al., 2020), it was unsurprising that A673 exhibited the highest average expression of these genes. To interrogate EwS TGs using chromatin-accessibility data, we generated gene activity scores (Granja et al., 2021) which model accessibility of regulatory elements in the vicinity of a given gene. These scores confirmed that chromatin accessibility of EwS TGs was higher in EwS cell lines compared to non-EwS cells (Figure 1—figure supplement 1D). To compare EWS::FLI1 motif enrichment across cells, we calculated single-cell ChromVAR deviation scores (Granja et al., 2021) of GGAA-microsatellites (four or more consecutive GGAA repeats; abbrev. ‘GGAA- µSat’) in accessible regions. As anticipated, this analysis revealed low enrichment in non-EwS cell lines. Unexpectedly, stark differences in EWS::FLI1 motif enrichment were observed across EwS cell lines (Figure 1B). In some cell lines accessibility of GGAA- µSats was similar across single cells (e.g. TC71 and RDES) while others showed a broader range of accessibility (e.g. SKNMC, TC32, and PDX305). CHLA10 cells showed a clear bimodal distribution of EWS::FLI1 motif accessibility (Figure 1B). This hints that within these cell lines, heterogeneity exists among individual cells with respect to accessibility of GGAA- µSats. Next, we collated a list of publicly available EWS::FLI1 fusion binding sites (Orth et al., 2022) with or without a GGAA µSat present. We then assessed chromatin accessibility at these published EWS::FLI1 binding sites across all cell lines and discovered that accessibility at these loci varied in a cell-line-specific manner (Figure 1C and Figure 1—figure supplement 1D).

Given the heterogeneity in the magnitude of EWS::FLI1 motif enrichment across EwS cell lines, we hypothesized that EwS cell lines differ in the proportion of cells expressing high levels of the EWS::FLI1 fusion transcript. It has been reported that lower levels of EWS::FLI1 gene expression and activity induce mesenchymal phenotypes in EwS cell lines (Apfelbaum et al., 2022a). To address this, we designed an approach to enrich EWS::FLI1 fusion transcript from the RNA components of our multiomic capture using biotinylated hybridization probes that tiled the EWSR1 and FLI1 gene loci. We then performed long-read sequencing on the product and counted fusion reads using novel fusion detection software designed specifically for long-reads (see Methods for details). As expected, EWS::FLI1 fusion transcripts were detected in EwS cells (Figure 1D). Scant non-EwS cells showed low levels of the fusion, likely from PCR artifacts of low-complexity products. Overall, however, we observed that EwS cell lines exhibit a high degree of variability in detectable EWS::FLI1 fusion transcript (Figure 1D and Figure 1—figure supplement 1E), possibly reflecting a difference in expression levels among individual cells. Generally, the proportion of cells with detectable transcript correlated with values obtained using single-cell qPCR to assess fusion expression (Franzetti et al., 2017). These findings are generally congruent with motif enrichment in that high fusion-expressing cell lines (e.g. A4573, SKNMC, and RDES) exhibit high EWS::FLI1 motif enrichment while cell lines bearing lower levels of fusion transcript and FLI1 product (e.g. PDX305, TC32, and CHLA9) exhibit lower EWS::FLI1 chromatin signature (Figure 1B, D, E). By contrast, other cell lines displayed inverse trends of fusion transcript and product to chromatin enrichment (e.g. A673 and TC71).

To profile the global chromatin and transcriptional heterogeneity using an unbiased approach, we calculated top differentially expressed (DE) genes and differentially accessible (DA) regions across EwS and non-EwS cells. As with the EwS TG-focused analysis, this approach showed that top DE/DA features readily distinguished EwS cells from non-EwS cells (Figure 1F, G). We observed self-aggregation of EwS cells into distinct clusters. Specifically, in terms of top DA regions, CHLA9, CHLA10, TC32, and PDX305 cells were more similar to each other than other EwS cell lines (Figure 1G). Altogether, these data show that EwS cell lines exhibit heterogeneous gene-regulatory profiles that segregate into groups that display distinct trends in accessibility, potentially reflecting unique underlying gene regulatory networks that are active in discrete cell contexts.

### EwS cells utilize gene regulatory programs that drive inter-tumoral heterogeneity

**Figure 2.**
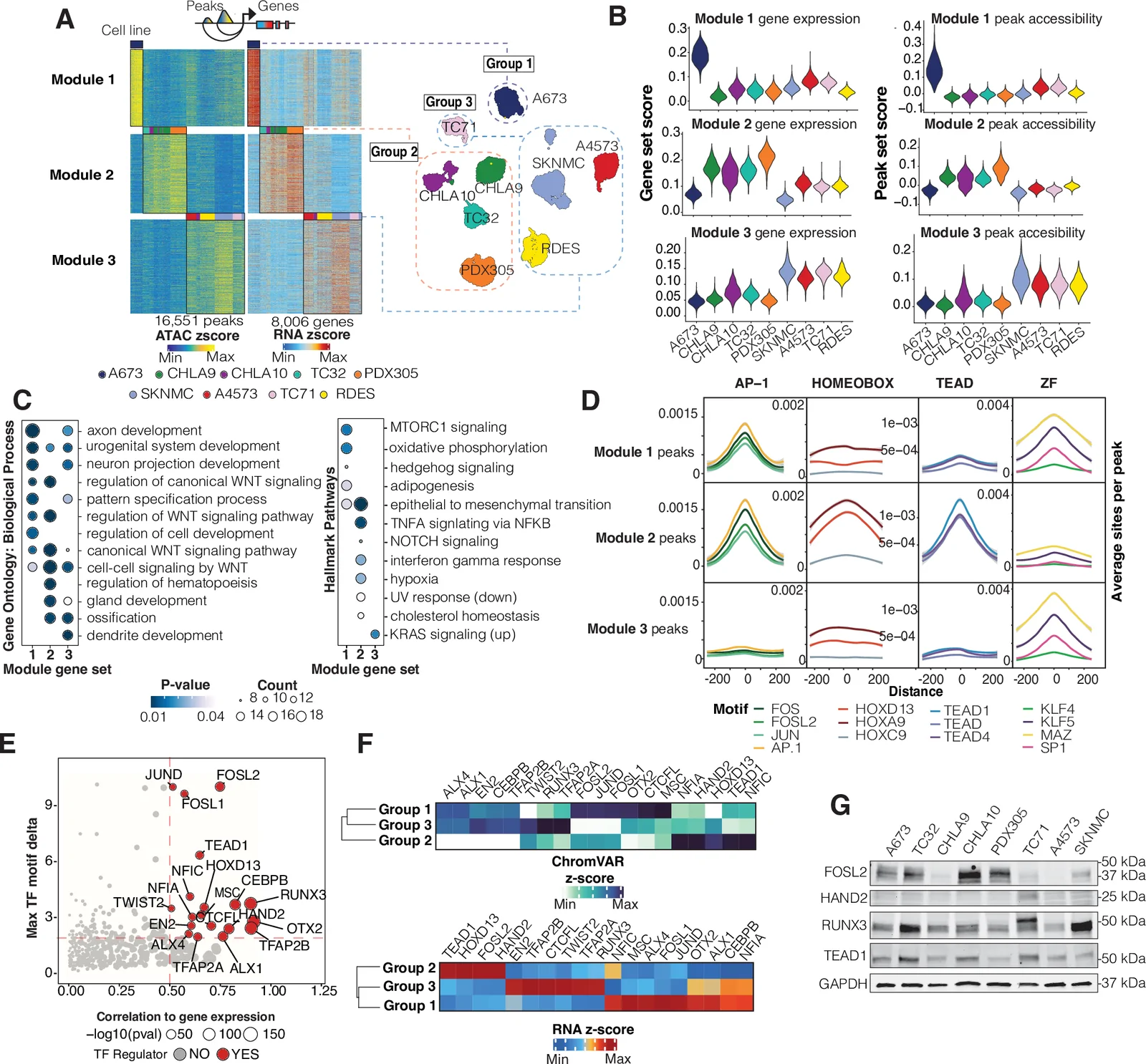
EwS cells utilize distinct gene regulatory modules. (**A**) Heatmap of unsupervised *k*-means clusters of peak-to-gene links (PGLs) across nine EwS cell lines. Left—peaks, right—genes, top—colors denote cell lines. Right of heatmap-UMAP of cell lines labeled by subgroup. Group 1 labeled with navy/dashed lines, group 2 in orange, and group 3 in light blue. (**B**) Violin plot of modules 1–3 gene expression and peak accessibility across EwS cell lines. (**C**) Universal enrichment analysis of module gene (top 300 filtered on peak-to-gene correlation) sets using both MSigDB Hallmark pathways and Gene Ontologies: Biological Process. The top 7 terms filtered by lowest p-value (those greater than 0.05 are not plotted) and count are shown. More significant terms are darker blue. The number of genes in each module that overlap with a gene set term is indicated by the count metric—represented as dot size. (**D**) HOMER de novo motif enrichment for each module peak set. Results depict the top significantly enriched motifs in each module peak set, and enrichments in other modules. (**E**) TFs with high expression and motif enrichment in the entire single-cell data set of EwS cell lines. Statistically significant TFs correlation to gene expression >0.5 and a maximum inter EwS subgroup difference in motif deviation *z*-score (denoted max TF motif delta) >1.9 highlighted in red. (**F**) ChromVAR motif deviation scores and gene expression of highly enriched TFs, parsed by EwS subgroup. (**G**) Western blot of selected TFs across EwS cell lines. FOSL2, HAND2, and RUNX3 were probed on one membrane and TEAD1 and GAPDH on a second membrane. GAPDH is shown as the loading control. Figure 2—source data 1.PDF file containing original western blots for Figure 2G, indicating the relevant bands and cell lines. Figure 2—source data 2.Original files for western blot analysis displayed in Figure 2G (FOSL2, HAND2, RUNX3, and vinculin from gel 1; TEAD1 and GAPDH from gel 2).

Next, to evaluate how cis-regulatory gene networks differ across EwS cells, we correlated transcript expression to regions of accessibility using the joint measurements of transcript count and accessibility in each cell (so-called ‘peak-to-gene linkages’; Granja et al., 2021). Hierarchical clustering of these cis-regulatory networks with a *k* parameter equal to 3 clearly separated CHLA9, TC32, and PDX305 into one distinct network module and SKNMC, A4573, RDES, and TC71 into another (Figure 2A), recapitulating what we observed using top DE/DA features (Figure 1F, G). We selected *k* = 3 to extend the two-cluster structure observed in Figure 1F, G, reasoning that A673 represented a clear outlier whose distinct regulatory program would be obscured at lower *k*. Cell subsets of CHLA10 separated into either cluster 2 or 3 (Figure 2A) but more cells had cumulative expression of module 2 genes (Figure 2B). We then assigned the nine cell lines into three groups based on their dominant module signature (Figure 2A, B), resulting in the assignment of A673 cells as group 1, CHLA9, CHLA10, TC32, and PDX305 cells as group 2, and SKNMC, A4573, TC71, and RDES as group 3 (Figure 2A). Enrichment analysis using Gene Ontology and Hallmark terms confirmed that the genes associated with these modules have functional associations with distinct cell behaviors (Figure 2C). While there was some degree of overlap, module 2 showed a relative enrichment in GO terms related to mesenchymal identity, and modules 1 and 3 had enriched neuronal identity markers. Genes involved in oxidative phosphorylation and targets of mTOR were uniquely enriched in module 1, possibly due to the presence of a BRAF^V600E^ mutation in A673 cells given the reported effects of this mutation on the mTOR pathway (Faustino et al., 2012; Kakkar et al., 2016; Prabowo et al., 2014).

Next, we investigated if the gene modules might harbor distinct TF binding sites. We performed differential motif enrichment using HOMER (Heinz et al., 2010) and found a strikingly well-delineated, and module-specific enrichment of specific TF families across the cis-regulatory networks (Figure 2D). The AP-1 family of motifs (FOS, FOSL2, JUN, and AP1), which has been suggested to form ternary complexes with EWS::FLI1 (Kim et al., 2006), enriched in modules 1 and 2, but not in module 3 (Figure 2D). Zinc Finger (ZF) TFs (KLF4, KLF5, MAZ, and SP1) were most enriched in modules 1 and 3 with limited evidence for activity in regulation of module 2 (Figure 2D). Module 2 peaks uniquely exhibited motif enrichment for developmental regulators from the Homeobox family of TFs (HOXD13 and HOXA9) and downstream transcriptional regulators of the Hippo and Wnt signal transduction pathways (TEAD1 and TEAD4). To corroborate these findings, we performed an analysis which correlates, at the single-cell level, TF binding motifs in accessible regions with gene expression (Figure 2E). This showed that TEAD1, HOXD13, and AP-1 TFs were highly enriched in group 2 cells (Figure 2E, F). Group 3 lacked motif enrichment for AP-1 TFs but showed robust enrichment in ALX1/4, RUNX3, CEBPB, and EN2 TFs (Figure 2E, F). We assessed protein levels of selected TF regulators by western blots to evaluate their heterogeneity across the cell lines. The protein abundance of these TFs exhibited substantial variability among the cells tested (Figure 2G). Together, these multimodal analyses of single cells uncovered at least two dominant gene regulatory circuits. One of these (module 2) displays mesenchymal/EMT-like functionality that is likely to be mediated by high activity of TEAD, AP-1, and HOXD13 TFs. The other (module 3) has enriched neural program functionality most likely mediated by ZF TFs and neural and neural crest development TFs such as RUNX3 and HAND2.

### EwS gene regulatory modules are differentially regulated by EWS::FLI1

**Figure 3.**
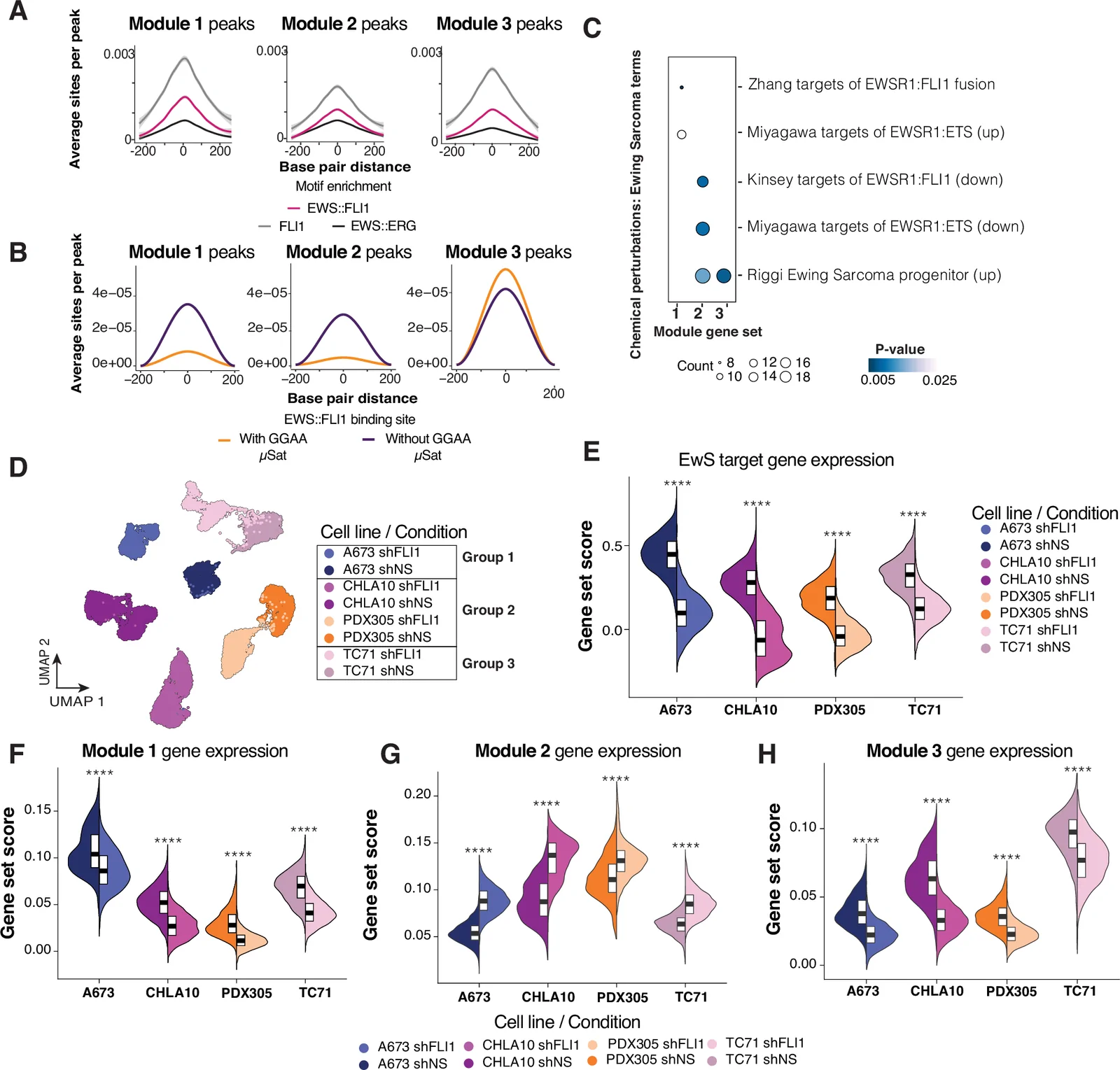
EwS gene regulatory modules are differentially regulated by EWS::FLI1. (**A**) HOMER enrichment of EWS/ETS motifs (EWS::FLI1, pink; FLI1, gray; EWS::ERG, black) in module peak sets. (**B**) HOMER enrichment of EWS::FLI1 binding sites, separated by regions with (orange) or without (dark purple) GGAA microsatellites. (**C**) Universal enrichment analysis of MSigDB EWS::FLI1 and Ewing sarcoma terms in module gene sets. (**D**) UMAP embeddings of shNS and shFLI1 samples. EwS subgroup identity of cell lines represented by dashed boxes. Group 1 (A673) labeled with navy/dashed lines, group 2 (CHLA10, PDX305) in orange, and group 3 (TC71) in light blue. (**E**) Expression of EwS target gene expression across shNS versus shFLI1 samples. (**F–H**) Module gene set expression across knockdown samples. ^****^ indicates p < 1 × 10^–4^ computed by a Wilcoxon rank-sum test.

We next sought to address whether the identified gene regulatory modules are directly or indirectly modulated by EWS::FLI1. We first measured EWS::FLI1, EWS::ERG (the second most common fusion in EwS tumors), and wild-type FLI1 motif enrichment and found all to be enriched in all three modules (Figure 3A), suggesting that EWS::FLI1 is likely to regulate expression of linked genes across all cells irrespective of the dominant module. However, by annotating these sites using published EWS::FLI1 binding sites (Orth et al., 2022) and segregating into those with or without a GGAA microsatellite (GGAA- µSat), we observed enrichment of GGAA- µSats only in module 3 peaks (Figure 3B). Thus, this analysis suggests that EWS::FLI1 binding is likely to directly influence gene regulatory circuits and its impact on transcriptional state depends on GGAA patterning, as has previously been suggested (Riggi et al., 2014).

**Figure 3—figure supplement 1.**
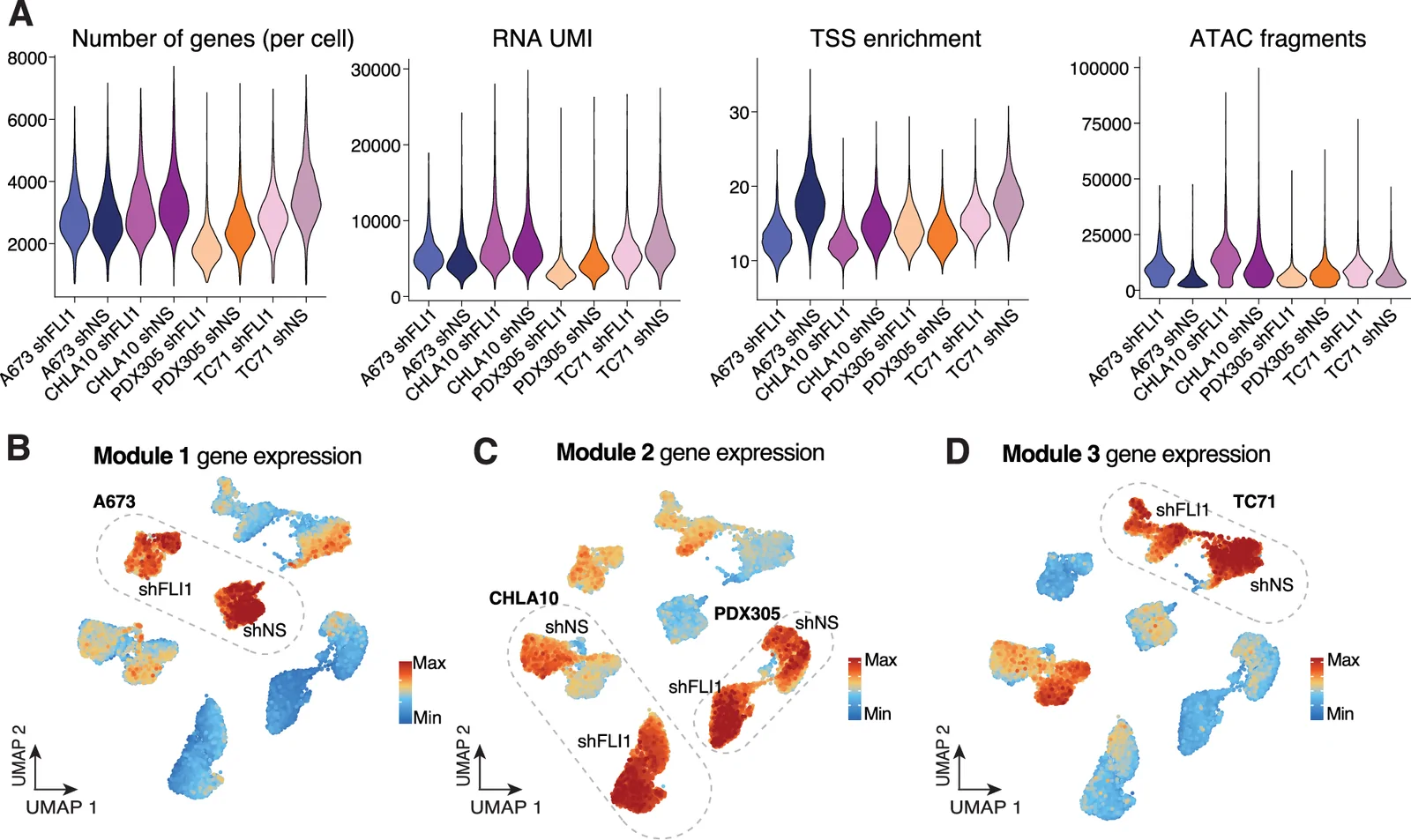
EwS gene regulatory modules are differentially regulated by EWS::FLI1. (**A**) Quality control metrics of knockdown samples. (**B–D**) UMAP embedding of gene expression scores of module gene sets. Cell lines most enriched for a module gene set are highlighted.

To begin to address the impact of EWS::FLI1 activity on cis-regulatory modules, we sourced data from published lists of EWS::FLI1 ‘activated’ and ‘repressed’ genes and performed gene set enrichment (Kinsey et al., 2006; Figure 3C). Overall, EWS::FLI activated genes were enriched in all modules, whereas repressed genes were enriched only in module 2 (Figure 3C). When combined with the lack of GGAA-µSat enrichment in module 2 (Figure 3B), these observations are consistent with previous reports that repressed loci usually do not have long stretches of GGAA repeats (Riggi et al., 2014; Johnson et al., 2017). Altogether, these data reveal distinct and polarized fusion-mediated regulatory events across EwS cell lines that differentially depend on GGAA repeats, suggesting that the diversity of fusion function is dependent on the motif landscape at fusion binding sites.

To directly address how different gene modules are affected by EWS::FLI1, we performed single-cell multiomic sequencing on selected cell lines from each EwS subgroup after shRNA-mediated knockdown of the fusion (Figure 3D). Following quality control (Figure 3—figure supplement 1A), we recovered 11,614 cells expressing negative control shRNA (A673 shNS, CHLA10 shNS, PDX305 shNS, and TC71 shNS) and 12,466 cells expressing shRNA specific to the FLI1 transcript (A673 shFLI1, CHLA10 shFLI1, PDX305 shFLI1, and TC71 shFLI1). All shFLI1 cells showed decreased expression of EwS TGs compared to shNS cells (Figure 3E), providing evidence that the knockdown effectively induced loss of EWS::FLI1 activity. EWS::FLI1 knockdown had specific, predictable, and reproducible effects on all cell lines. Importantly, the same stratification of shNS cell lines into groups with specific module enrichment was observed, indicating that lentiviral transduction and selection did not alter dominant gene programs (Figure 3F-H, Figure 3—figure supplement 1B-D). In addition, each module was affected by EWS::FLI1 knockdown in a manner consistent with predictions based on module gene sets. Specifically, EWS::FLI1 knockdown resulted in decreased expression of module 1 and 3 genes (Figure 3F–H, Figure 3—figure supplement 1B–D) and increased expression of module 2 genes (Figure 3G, Figure 3—figure supplement 1C). These data confirm that EWS::FLI1 activates cis-regulatory networks found in modules 1 and 3 but represses module 2. The results also support our initial hypothesis that EWS::FLI1-regulated gene modules differ among commonly used EwS cell lines. They further show that specific and reproducible patterns of transcriptomic and phenotypic signatures dominate each cell line, and these predictably respond to fusion knockdown.

### CHLA10 exhibits intra-tumoral heterogeneity of gene regulatory modules

**Figure 4.**
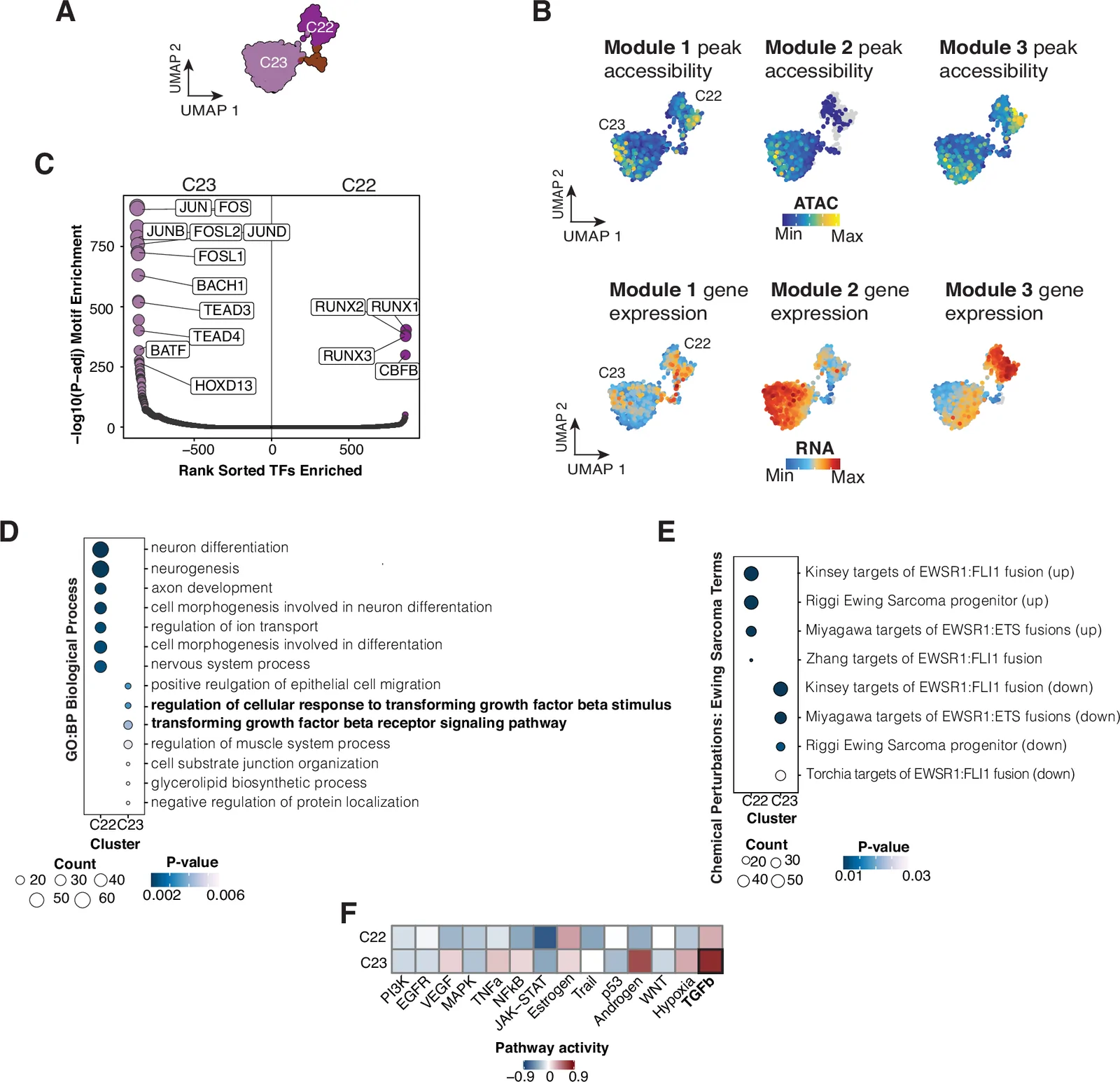
CHLA10 is a microcosm of EWS::FLI1-mediated heterogeneity. (**A**) UMAP of CHLA10 colored by cluster, C22 dark purple, C23 light purple. (**B**) UMAP of module peak accessibility and gene set expression in CHLA10 cells. (**C**) Differential motif enrichment between C22 and C23, TFs with −log_10_(p-value) >250 highlighted. (**D**) Universal enrichment of GO: Biological Process terms in differentially expressed genes between C22 and C23, terms associated with TGF-β in bold. (**E**) Universal enrichment of MSigDB Ewing Sarcoma terms in differentially expressed genes between C22 and C23. (**F**) Pathway activity scores across C22 and C23, TGF-β highlighted in bold.

Most EwS cell lines in this study are dominated by a single gene-regulatory program (module 1, 2, or 3). A notable exception is CHLA10, which contains mutually exclusive sub-programs defined by differences in both EWS::FLI1 motif activity and module utilization (Figures 1B and 2A). This distinction is particularly striking because CHLA10 and CHLA9 originated from tumors from the same patient, with CHLA10 being derived post-therapy and following disease relapse. To further investigate this intratumoral heterogeneity, we performed unsupervised Louvain clustering (Stuart et al., 2019) on EwS cell lines (Figure 4—figure supplement 1A) and computed the Pearson correlation coefficient of the most highly variable regions of accessibility. As expected, CHLA9, TC32, and PDX305 cells grouped together based on similarity (Figure 4—figure supplement 1B). In contrast, CHLA10 segregated into two clusters (C22 and C23) that each showed greater transcriptional and epigenetic similarity to other cell lines than to each another (Figure 4A, Figure 4—figure supplement 1B), highlighting the extent of its internal heterogeneity. Unsurprisingly, of all cell lines, CHLA10 C23 cells exhibited the greatest correlation to profiles from CHLA9.

**Figure 4—figure supplement 1.**
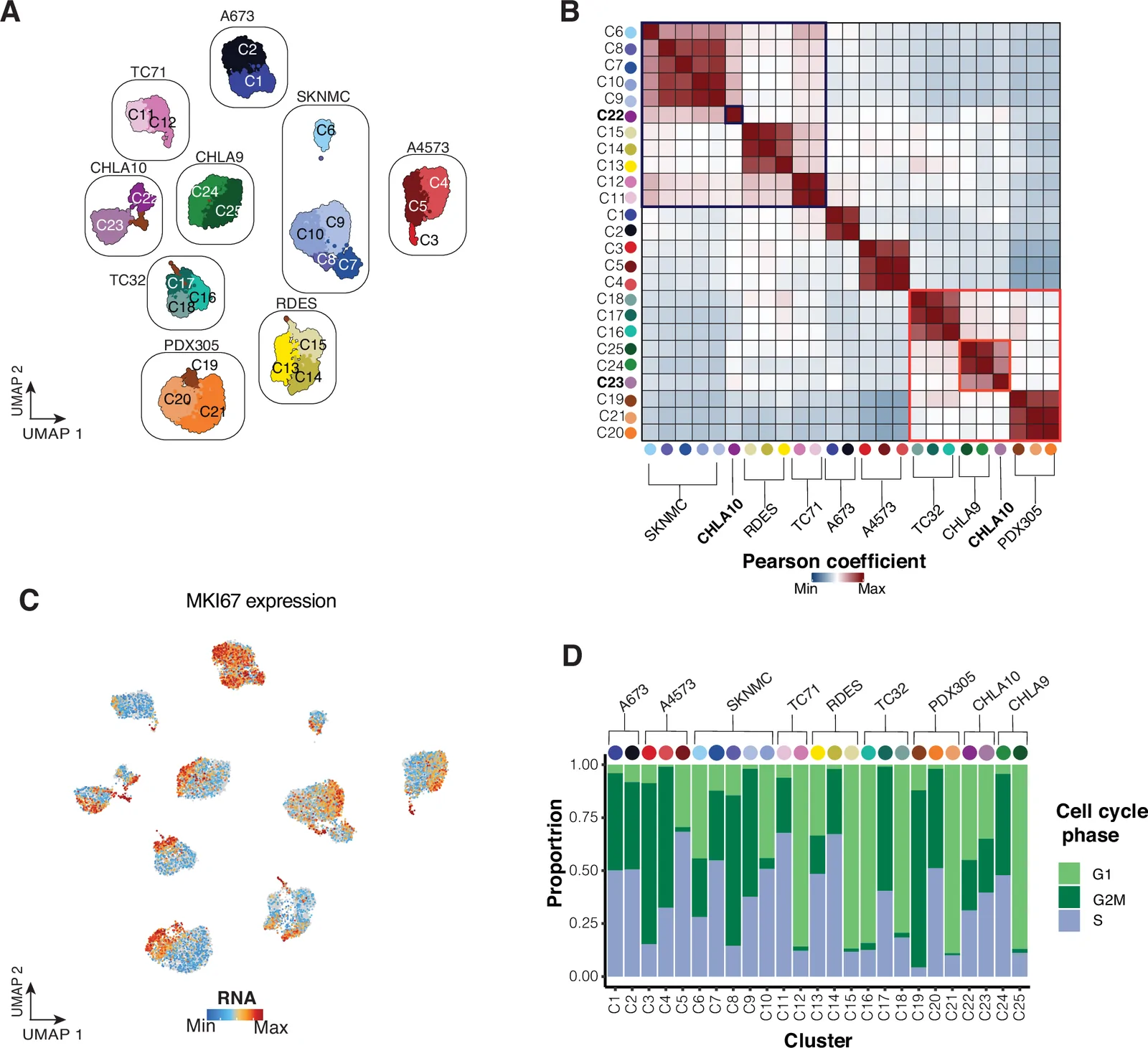
CHLA10 is a microcosm of EWS::FLI1-mediated heterogeneity. (**A**) UMAP of nine EwS cell lines colored and labeled by cluster (**B**) Pearson correlation heatmap of EwS clusters. The rows are labeled by cluster ID, the columns are labeled by cell line identity. Cell lines sharing epigenetic signatures highlighted in bold and red boxes. (**C**) *MKI67* expression across EwS cell lines. (**D**) Proportion of cells in G1, G2M, and S phase within EwS clusters.

Cell-cycle effects contributed to but could not fully explain CHLA10 heterogeneity (Figure 4—figure supplement 1C, D). Instead, plotting peak accessibility and transcript abundance of each module revealed that C22 cells enriched for module 3 while C23 cells relied on module 2 (Figure 4B). Motif enrichment of DA peaks further underscored the nature of the split: RUNX motifs were enriched in C22 cells, whereas AP-1, TEAD, and HOX TF motifs predominated in C23 cells (Figure 4C). Gene-set enrichment mirrored these findings; C22 expressed neural programs, whereas C23 cells were enriched for EMT, migration, and TGF-β signaling gene sets (Figure 4D, E). Pathway-activity inference analysis between cell clusters (Badia-I-Mompel et al., 2022) further implicated TGF-β as a dominant upstream driver in C23 (Figure 4F).

Collectively, the isogenic model CHLA9/CHLA10 encapsulates the spectrum of transcriptional states seen across the other EwS cell lines and pinpoints TGF-β signaling as a key mediator of the module 2 transcriptional state.

### Sub-populations of EwS cell lines differentially respond to TGF-β stimulus

**Figure 5.**
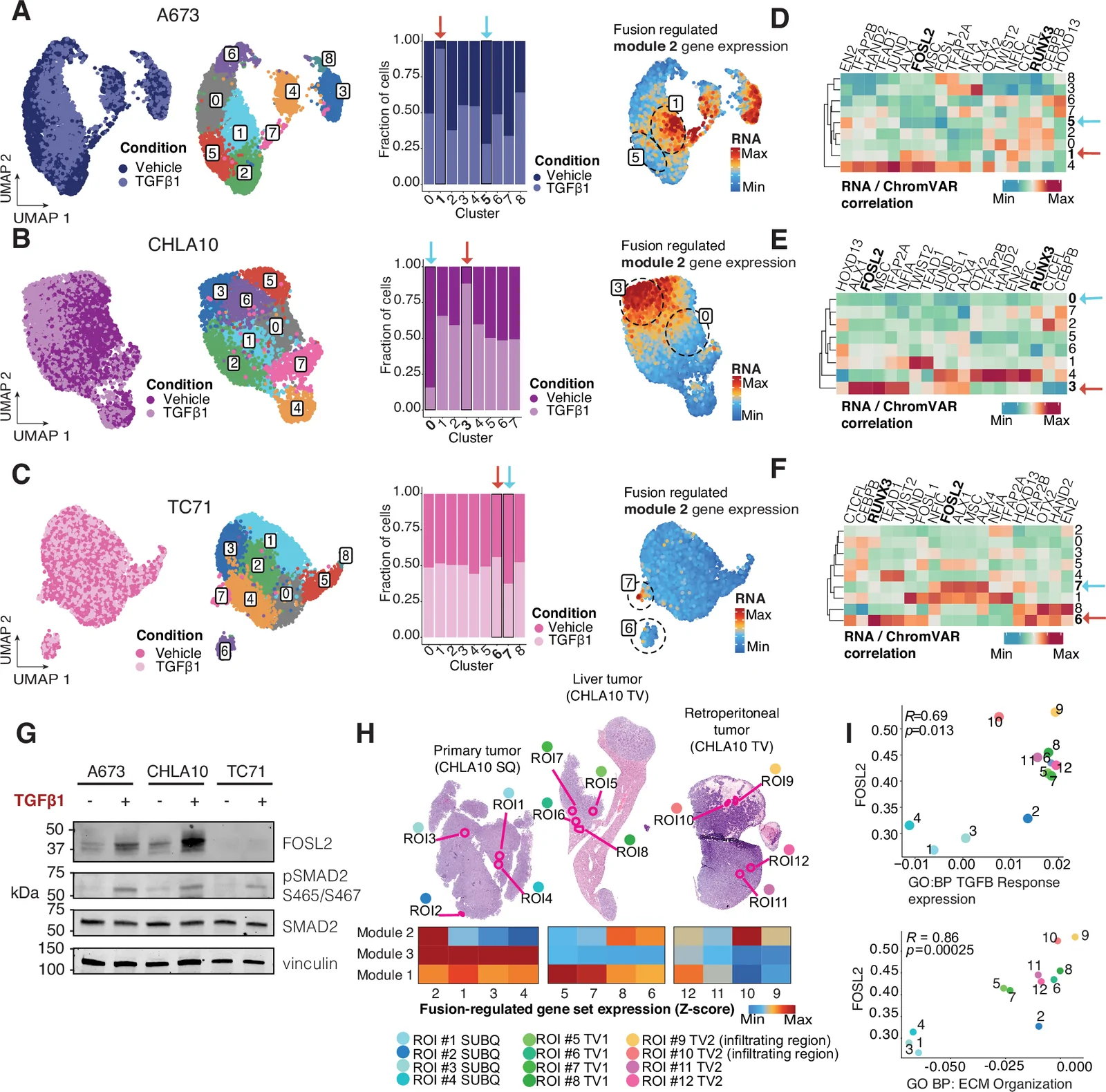
Sub-populations of EwS cell lines differentially respond to TGF-β stimulus. (**A–C**) UMAP of A673, CHLA10, and TC71 cells colored by condition (vehicle control or TGF-β1 treatment)*—column 1*; and cluster*—column 2*; fraction of treated versus control cells in each cluster with highest and lowest indicated by arrow*—column 3*; expression of fusion regulated module 2 gene set, highest and lowest TGF-β clusters highlighted with dashed lines—*column 4*. (**D–F**) Correlation between TF expression and chromVAR TF deviation scores of EwS TFs across clusters, highest (red) and lowest (blue) TGF-β clusters highlighted by arrows. (**G**) Western blot of active (pS465/467) and total SMAD2 and FOSL2 protein in A673, CHLA10, and TC71 cells after 24 hr TGF-β1 treatment. (**H**) H&E staining of 3 CHLA10 xenografts in NSG mice generated by subcutaneous or tail vein injection (*upper left*). Those regions of interest (ROIs) subjected to whole human transcriptome digital spatial profiling are highlighted. Heatmap of module gene expression across ROIs (*bottom left*). (**I**) Scatter plot of average expression of Gene Ontology: Biological Process (GO:BP) TGF-β response genes and extracellular matrix (ECM) organization genes compared to *FOSL2* expression in ROIs. Pearson R and p-value highlighted (*top left*). Figure 5—source data 1.PDF file containing original western blots for Figure 5G, indicating the relevant bands and treatments. Figure 5—source data 2.Original files for western blot analysis displayed in Figure 5G (FOSL2, phospho-SMAD2 S465/467, SMAD2, and vinculin).

Prior reports have shown that TGF-β promotes pro-angiogenic extracellular matrix (ECM) gene expression in EwS (Hawkins et al., 2020). To interrogate the impact of TGF-β signaling on the newly defined transcriptional states in EwS, we performed multiomic profiling on A673 (module 1-dominant), CHLA10 (module 2 dominant), and TC71 (module 3 dominant) after treatment with recombinant TGF-β1 for 24 hr and compared them to vehicle-treated controls. After quality control filtering, 19,611 cells were retained for analysis (Figure 5—figure supplement 1A). Dimensionality reduction revealed substantial shifts in both gene expression and chromatin accessibility in A673 and CHLA10 following TGF-β treatment, whereas TC71 profiles remained relatively stable (Figure 5A–C). In A673 and CHLA10, TGF-β treatment resulted in a clear polarization of cells toward module 2 usage (Figure 5A–C), while expression patterns of modules 1 and 3 remained consistent with prior observations (Figure 5—figure supplement 1B). The muted response of the TC71 cell line (module 3 dominant) to the influence of TGF-β (Figure 5A–C, Figure 5—figure supplement 1B) suggests that pre-existing transcriptional states may condition the sensitivity to TGF-β signaling.

**Figure 5—figure supplement 1.**
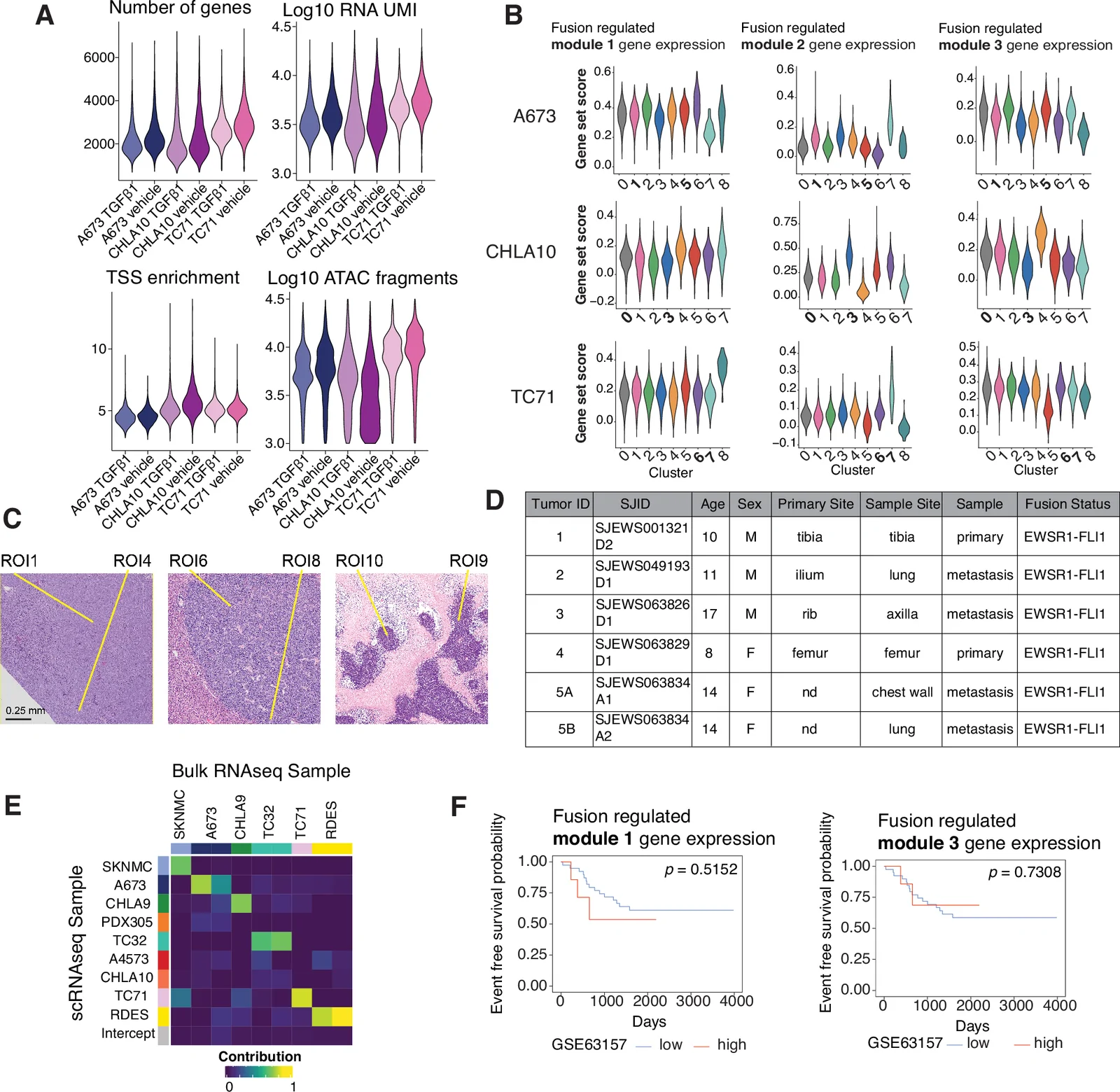
Sub-populations of EwS cell lines differentially respond to TGF-β stimulus. (**A**) Quality control metrics of vehicle control and TGF-β treated cell lines. (**B**) UMAP colored by gene expression of fusion regulated modules 1 and 3 across cell lines. (**C**) Zoomed-in image of regions of interest (ROIs) 1, 4, 6, 8, 9, and 10. (**D**) Clinical details of patient samples subjected to single-cell sequencing. (**E**) Heatmap depicting similarity scores of publicly available bulk EwS cell line data with multiome scRNA-seq data. (**F**) Survival curves of patient microarray data (Volchenboum et al., 2015) (*n* = 46), high expression of module genes 1 and 3 set in red (top 15% expressing samples, *n* = 7), low expression in blue (bottom 85%, *n* = 39).

We next examined TF expression and motif accessibility in clusters exhibiting the most pronounced or lowest enrichment of TGF-β-treated cells (indicated by arrows in Figure 5A–F). Correlation analyses revealed a reciprocal enrichment pattern of many key TFs from Figure 2, where correlation of FOSL2 (but not RUNX3) gene expression and accessibility was generally highest in TGF-β responsive clusters. Western blot validation confirmed TGF-β-induced FOSL2 expression specifically in CHLA10 but not in the relatively insensitive TC71 cells, despite observed SMAD2 phosphorylation (Figure 5G).

Our recent whole human transcriptome digital spatial profiling (DSP) study of CHLA10 tumor xenografts (Nanostring GeoMx) showed that transcriptionally distinct tumor cell subpopulations are spatially distributed (Wrenn et al., 2023). We therefore interrogated these data to determine if the identified gene regulatory modules in CHLA10 cells were randomly or spatially distributed across tumors in vivo. We first selected genes from modules 1 and 3 that were significantly upregulated (average log_2_ fold change >0.25) by EWS::FLI1 as well as module 2 genes that were significantly repressed by the fusion (average log_2_ fold change <–0.25) and applied these fusion-regulated gene sets to the DSP data. Intriguingly, regions of interest (ROIs) that were captured from tumor borders (ROIs 2, 6, 8, and 10) were enriched with tumor cells that displayed heightened module 2 expression, whereas densely packed tumor cores (ROIs 1, 3, 4, 5, 7, and 11) favored modules 1 and 3 (Figure 5H, Figure 5—figure supplement 1C). Module 3 was also highly favored in subcutaneous compared to tail vein-derived disseminated tumors (Figure 5H). Cells captured specifically from the border of a metastatic retroperitoneal tumor (ROIs 9 and 10) expressed the highest levels of *FOSL2*, TGF-β-response genes, and ECM-organization programs (Figure 5I).

In summary, these findings highlight TGF-β-driven activation of module 2 gene expression in EwS cells via AP-1 TFs, particularly FOSL2. Furthermore, spatial analysis suggests module 2 programs are enriched at invasive tumor regions, underscoring the role of TGF-β signaling in mediating EwS cell heterogeneity and tumor progression dynamics.

### Patient tumors show heterogeneous usage of EwS cis gene regulatory modules

**Figure 6.**
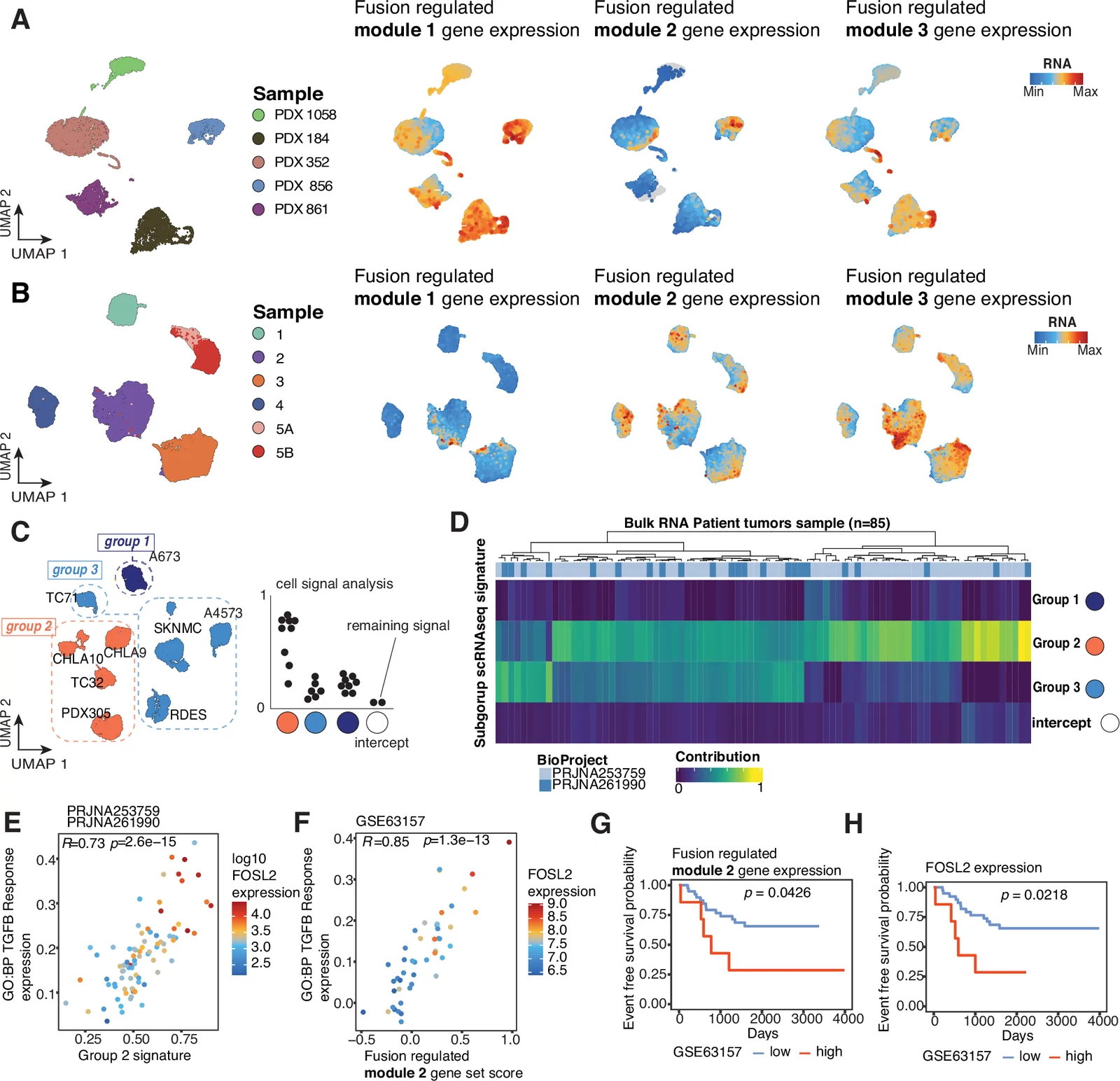
Human tumor data show usage of EWS::FLI1 gene regulatory modules. (**A**) UMAP embedding of five EwS PDX samples (*left*) UMAP of fusion regulated module gene sets in PDX samples (*right*). (**B**) UMAP embedding of single nuclei sequencing of 6 patient tumor samples (left). Patient tumor scRNA expression of fusion regulated modules (*right*). (**C**) Schematic depicting deconvolution method. (**D**) Heatmap EwS subgroup signal in bulk EwS patient tumor RNA-seq. The intercept row represents remaining signal (bottom). Patient tumor samples annotated by GEO BioProject. (**E**) Scatter plot of bulk RNA patient samples by group 2 signature and GO: BP TGF-β response genes and colored log_10_ FOSL2 expression. Pearson R and p-value highlighted (*top left*). (**F**) Scatter plot of microarray patient samples by module 2 expression and GO: BP TGF-β response genes and colored FOSL2 expression. Pearson R and p-value highlighted (*top left*). (**G**) Survival curves of patient microarray data (Volchenboum et al., 2015; *n* = 46), high expression of module 2 gene set in red (top 15% expressing samples, *n* = 7), low expression in blue (bottom 85%, *n* = 39). (**H**) Survival curves of high expression of *FOSL2* samples (top 15%, *n* = 7, bottom 85%, *n* = 39).

We next sought to determine whether the gene-regulatory networks defined in our cell line panel are similarly deployed across the landscape of early passage patient-derived xenograft (PDX) and primary patient biopsies. To determine how the three regulatory gene programs manifest in these tumors, we used the fusion-regulated gene sets as defined above. In a series of publicly available PDX tumor profiles (Aynaud et al., 2020), module 1 was prominent, while modules 2 and 3 (Figure 6A) showed more modest and heterogeneous enrichment. On the other hand, in six primary EwS tumor samples (Hawkins et al., 2026; Figure 6B, Figure 5—figure supplement 1D), profiled by single-nucleus RNA-seq, every case contained discrete subpopulations that preferentially expressed either module 2 or 3. In contrast to the PDX data, module 1-positive cells were comparatively rare in primary tumors.

We next asked whether these single-cell patterns could be detected in bulk transcriptome data that were generated from three independent patient datasets (Crompton et al., 2014; Brohl et al., 2014; Volchenboum et al., 2015). To achieve this we employed a deconvolution method (Young et al., 2021) that identifies the overlap of signals from bulk measurements with a single-cell transcriptomic reference and captures any proportion of the bulk signal not represented by single-cell data in an ‘intercept’ term (schematized in Figure 6C). We first validated the method on bulk RNA sequencing data taken from many of the same EwS cell lines we profiled in our experiments and found a high level of concordance with little signal captured in the intercept term (Figure 5—figure supplement 1E). We then tested the transcriptomic overlap of EwS subgroups in 85 non-clinically annotated patient tumors that were profiled by bulk RNA sequencing in two independent cohorts (PRJNA253759 and PRJNA261990). Group 2 signals were evident in nearly all tumors and group 3 cell signals were also present in more than half of tumors, confirming heterogeneity of gene network usage and cell state in primary tumors (Figure 6D). Tumors with abundant group 2 signal also displayed significantly higher expression of TGF-β response genes and FOSL2 expression (Figure 6E), linking our in-vitro observation that TGF-β drives module 2 activation to patient tumors in the clinical setting.

Finally, we examined microarray data from a series of 46 clinically annotated primary tumors (GSE63157) that were uniformly treated and for whom outcome data were available (Volchenboum et al., 2015). First, we observed significant correlation between expression of FOSL2, TGF-β response and module 2 expression (Figure 6F). Furthermore, high expression of fusion-repressed module 2, and particularly of its key effector *FOSL2*, identified patients with markedly worse event-free survival, whereas modules 1 and 3 had little prognostic value (Figure 6G, H, Figure 5—figure supplement 1F). Module 2, linked to TGF-β responsiveness and FOSL2 activity, emerges as the predominant—and clinically adverse—program in primary EwS, underscoring its potential as both a biomarker of aggressive disease and a therapeutic target.

## Discussion

Single-cell genomics has made it possible to chart, with unprecedented resolution, the transcriptional circuitry that underlies intratumoral diversity (Gavish et al., 2023; Marusyk et al., 2012). In EwS, a cancer that has very few genetic mutations beyond its canonical chromosomal translocation (Aynaud et al., 2020; Apfelbaum et al., 2022b; Wrenn et al., 2023; Apfelbaum et al., 2022a) and notable clinical heterogeneity, this diversity likely arises from epigenetic plasticity (van Groningen et al., 2017; Boeva et al., 2017). Our study enhances this understanding by defining two distinct transcriptional and chromatin accessibility states consistently observed across cell lines, PDXs, and primary tumor samples.

The first transcriptional state, associated with module 3 genes, is enriched for canonical GGAA-microsatellite enhancers, high EWS::FLI1 expression, and neural-crest TFs such as TFAP2A and RUNX3. In contrast, the second state associated with module 2 genes lacks GGAA repeats, is suppressed by EWS::FLI1, and involves activation by TEAD and AP-1 family members together with the homeobox factor HOXD13—all elements previously linked to epithelial-to-mesenchymal transition (EMT), invasion, and a low-fusion state (Zhang et al., 2009; Katschnig et al., 2017; Croushore et al., 2024).

The mutually antagonistic architecture of these modules is consistent with earlier observations that EWS::FLI1-repressed loci tend to be short-repeat or single ETS sites that recruit additional repressor complexes such as ETV6 (Riggi et al., 2014; Sankar et al., 2013; Lu et al., 2023.) Further supporting this, recent data Sanalkumar et al., 2023 demonstrate EWS::FLI1’s role in shaping cell identity by controlling promoter-enhancer looping and 3D chromatin networks. Loss of EWS::FLI1 was shown to alter 3D chromatin structure, activating enhancers enriched for AP-1 motifs and facilitating mesenchymal gene expression, consistent with our findings.

Analysis of three independent patient cohorts confirmed module 2 and 3 signatures in most primary tumors. Clinically, elevated expression of module 2 genes, notably FOSL2, correlated with inferior event-free survival in a prospectively treated cohort of patients with localized EwS. This association nominates module 2 expression as a potential biomarker of aggressive disease and links TGF-β signaling to EMT-like transcriptional states and poor prognosis.

Another key insight from our study is the link between cells expressing high levels of module 2 genes and heightened responsiveness to TGF-β. These findings argue that external cues can re-wire specific subsets of EwS cells toward a more extreme mesenchymal/EMT-like state in the absence of genetic change. This is in line with emerging data that cell plasticity in EwS is influenced by a TGF-β-induced transition of EwS cells to cancer-associated fibroblast-like states (Wrenn et al., 2023), which appear to be ‘unlocked’ by TGFB2 response (Wrenn et al., 2025). Thus, therapeutic strategies that disrupt these microenvironmental signals—or directly target downstream effectors such as FOSL2—may limit metastatic spread by constraining module 2 activation. Moreover, by promoting a fibroblast-like phenotype and dampening inflammatory signaling, TGF-β may also contribute to immune evasion and the establishment of an immunosuppressive tumor microenvironment (Visser et al., 2023; Goodspeed et al., 2025; Kumar et al., 2025).

Our study is constrained by the limited number of primary single-cell datasets currently available; larger, clinically annotated cohorts will be required to validate the prognostic utility of module balance and to map temporal dynamics during therapy and relapse. Moreover, while CHLA9/CHLA10 serves as a useful isogenic model of plasticity, early-passage or organoid systems may preserve an even richer landscape of states. Finally, dissecting the full spectrum of extrinsic factors—beyond TGF-β—that tip the balance between modules will be key to developing microenvironment-targeted interventions.

In summary, our study provides novel insights into the TF regulatory networks that interact with the EWS::FLI1 fusion protein to sustain distinct cellular states and facilitate dynamic transitions between these states. Specifically, we identified core gene network modules and key TFs, notably FOSL2 and other module 2 TFs, which antagonize fusion-mediated repression, promoting a mesenchymal and EMT-like state associated with more aggressive disease phenotypes. Furthermore, our findings highlight cell-extrinsic TGF-β as a critical factor capable of activating FOSL2 and driving the aggressive module 2 program in vivo. By uncovering these regulatory mechanisms and their functional implications, we offer a foundation for developing therapeutic strategies aimed at limiting metastasis, overcoming treatment resistance, and ultimately improving clinical outcomes for patients with EwS.

## Materials and methods

**Key resources table:** 

Reagent type (species) or resource	Designation	Source or reference	Identifiers	Additional information
Gene (*Homo sapiens*)	EWSR1	NCBI Gene	Gene ID: 2130; HGNC:3508	5′ fusion partner of the EWS::FLI1/EWS::ETS oncogene
Gene (*Homo sapiens*)	FLI1	NCBI Gene	Gene ID: 2313; HGNC:3749	3′ ETS-family fusion partner; target of shRNA knockdown
Gene (*Homo sapiens*)	FOSL2	NCBI Gene	Gene ID: 2355; HGNC:3798	Key module 2 (AP-1) effector linked to TGF-β response
Cell line (*Homo sapiens*)	A-673 (A673)	ATCC	RRID:CVCL_0080	Ewing sarcoma; module 1 dominant; harbors BRAF V600E
Cell line (*Homo sapiens*)	SK-N-MC (SKNMC)	ATCC	RRID:CVCL_0530	Ewing sarcoma; high EWS::FLI1 motif/fusion
Cell line (*Homo sapiens*)	RD-ES (RDES)	ATCC	RRID:CVCL_2169	Ewing sarcoma; maintained in 15% FBS
Cell line (*Homo sapiens*)	TC-32 (TC32)	COG cell line repository	RRID:CVCL_7151	Ewing sarcoma; module 2 group
Cell line (*Homo sapiens*)	TC-71 (TC71)	COG cell line repository	RRID:CVCL_2213	Ewing sarcoma; module 3 dominant
Cell line (*Homo sapiens*)	A4573	COG cell line repository	RRID:CVCL_6245	Ewing sarcoma; high fusion expression
Cell line (*Homo sapiens*)	CHLA-9	COG cell line repository	RRID:CVCL_M150	Ewing sarcoma; isogenic pair with CHLA-10 (same patient, at diagnosis)
Cell line (*Homo sapiens*)	CHLA-10	COG cell line repository	RRID:CVCL_6583	Ewing sarcoma; isogenic with CHLA-9 (post-therapy/relapse); intratumoral heterogeneity model
Cell line (*Homo sapiens*)	PDX305	Wrenn et al., 2023	N/A	Ewing sarcoma; derived from a recurrent metastatic tumor; low fusion
Cell line (*Homo sapiens*)	U-2 OS (U2OS)	ATCC	RRID:CVCL_0042	Osteosarcoma; non-EwS control
Cell line (*Homo sapiens*)	RD	ATCC	RRID:CVCL_1649	Rhabdomyosarcoma; non-EwS control
Cell line (*Homo sapiens*)	5046L hMSC	Gift of Dr. Darwin Prockop	N/A	Human mesenchymal stem cell; non-EwS control
Cell line (*Homo sapiens*)	240L hMSC	Gift of Dr. Darwin Prockop	N/A	Human mesenchymal stem cell; non-EwS control
Cell line (*Homo sapiens*)	H7 hMSC	Gift of Dr. Alejandro Sweet-Cordero	N/A	Human mesenchymal stem cell; non-EwS control
Cell line (*Homo sapiens*)	293FT	Thermo Fisher Scientific	RRID:CVCL_6911; R70007 (Thermo Fisher)	Lentiviral packaging cell line
Biological sample (*Homo sapiens*)	Primary EwS patient tumor biopsies	St. Jude Children’s Research Hospital (ref 49)	N/A	Six primary tumors profiled by single-nucleus RNA-seq
Biological sample (*Homo sapiens*)	CHLA-10 xenograft tumors (GeoMx DSP)	Prior work (ref 21)	N/A	Whole-transcriptome digital spatial profiling of subcutaneous and disseminated tumors
Antibody	FLI1 (EPR4646) (Rabbit monoclonal)	Abcam	Cat# ab133485; clone EPR4646; RRID:AB_2722650	(WB), 1:1000
Antibody	Fra2/FOSL2 (D2F1E) (Rabbit monoclonal)	Cell Signaling Technology	Cat# 19967; clone D2F1E; RRID:AB_2722526	(WB)
Antibody	HAND2 (Goat polyclonal)	R&D Systems	Cat# AF3876; RRID:AB_2295155	(WB)
Antibody	RUNX3/AML2 (D9K6L) (Mouse monoclonal)	Cell Signaling Technology	Cat# 13089; clone D9K6L; RRID:AB_2798118	(WB)
Antibody	TEAD1 (D9X2L) (Rabbit monoclonal)	Cell Signaling Technology	Cat# 12292; clone D9X2L; RRID:AB_2797873	(WB)
Antibody	Phospho-SMAD2 (Ser465/467) (138D4) (Rabbit monoclonal)	Cell Signaling Technology	Cat# 3108; clone 138D4; RRID:AB_490941	(WB)
Antibody	SMAD2 (L16D3) (Mouse monoclonal)	Cell Signaling Technology	Cat# 3103; clone L16D3; RRID:AB_490816	(WB)
Antibody	GAPDH (6C5) (Mouse monoclonal)	Invitrogen/Thermo Fisher Scientific	Cat# AM4300; clone 6C5; RRID:AB_2536381	(WB)
Antibody	Vinculin (E1E9V) (Rabbit monoclonal)	Cell Signaling Technology	Cat# 13901; clone E1E9V; RRID:AB_2728768	(WB)
Antibody	IRDye 800CW Goat anti-Mouse IgG (H+L), highly cross-adsorbed	LI-COR Biosciences/LICORbio	Cat# 926-32210; RRID:AB_621842	Secondary antibody; WB; 1:10,000 in Intercept (TBS) Blocking Buffer + 0.1% Tween-20
Antibody	IRDye 680RD Goat anti-Rabbit IgG (H+L), highly cross-adsorbed	LI-COR Biosciences/LICORbio	Cat# 926-68071; RRID:AB_10956166	Secondary antibody; WB; 1:10,000 in Intercept (TBS) Blocking Buffer + 0.1% Tween-20
Antibody	IRDye 680RD Donkey anti-Goat IgG (H+L), highly cross-adsorbed	LI-COR Biosciences/LICORbio	Cat# 926-68074; RRID:AB_10956736	Secondary antibody; WB; 1:10,000 in Intercept (TBS) Blocking Buffer + 0.1% Tween-20; used for HAND2 (R&D Systems AF3876)
Recombinant DNA reagent	pLKO.1 shNS (non-targeting control shRNA) (plasmid)	Sigma-Aldrich (MISSION)	Cat# SHC002	Negative-control shRNA for fusion-knockdown multiome experiments
Recombinant DNA reagent	pLKO.1 shFLI1 (plasmid)	Sigma-Aldrich (MISSION)	Cat# TRCN0000005322	shRNA targeting the FLI1 transcript for EWS::FLI1 knockdown
Recombinant DNA reagent	pCD/NL-BH*DDD (packaging plasmid)	Addgene	RRID:Addgene_17531	Lentiviral packaging
Recombinant DNA reagent	pMD2.G (envelope plasmid)	Addgene	RRID:Addgene_12259	VSV-G envelope for lentivirus production
Sequence-based reagent	EWS::FLI1 hybridization capture probes (biotinylated)	IDT	/5Biosg/AGAACACCTATGGGCAACCG- AGCAGCTATGGACAGCAGAGTAGCT- ATGGTCAACAAAGCAGCTATGGGCA- GCAGCCTCCCACTAGTTACCaaaa /5Biosg/ATATGGACAGAGTAACTACA- GTTATCCCCAGGTACCTGGGAGCTA- CCCCATGCAGCCAGTCACTGCACCT- CCATCCTACCCTCCTACCAGaaaa /5Biosg/CCAGGCCTCCTATGCAGCTC- AGTCTGCATATGGCACTCAGCCTGC- TTATCCAGCCTATGGGCAGCAGCCA- GCAGCCACTGCACCTACAAGaaaa /5Biosg/GAAGCGGGCAGATCCAGCT- GTGGCAATTCCTCCTGGAGCTGCT- CTCCGACAGCGCCAACGCCAGCTG- TATCACCTGGGAGGGGACCAACGaaaa /5Biosg/AATGACGGACCCCGATGAG- GTGGCCAGGCGCTGGGGCGAGCG- GAAAAGCAAGCCCAACATGAATTAC- GACAAGCTGAGCCGGGCCCTCCGaaaa /5Biosg/ATAAAAACATTATGACCAAA- GTGCACGGCAAAAGATATGCTTAC- AAATTTGACTTCCACGGCATTGCC- CAGGCTCTGCAGCCACATCCGAaaaa-	Biotinylated probes tiling EWSR1 exons 5–7 and FLI1 exon 9 for long-read fusion enrichment
Peptide, recombinant protein	Recombinant human TGF-β1	R&D Systems	Cat# 7754-BH-005	10 ng/ml in 4 mM HCl/0.1% BSA; 24 hr treatment
Commercial assay or kit	Chromium Next GEM Single Cell Multiome ATAC + Gene Expression Kit	10x Genomics	Chromium Next GEM Single Cell Multiome ATAC +Gene Expression Reagent Bundle, PN-1000283; protocol CG000338	Joint scRNA + scATAC library preparation
Commercial assay or kit	xGen Hybridization and Wash v2 kit	Integrated DNA Technologies (IDT)	Cat# 10010351	Target enrichment of EWS::FLI1 fusion cDNA
Commercial assay or kit	SMRTbell Prep Kit 3.0	Pacific Biosciences (PacBio)	Cat# 102-141-700	Long-read library preparation
Commercial assay or kit	GeoMx Digital Spatial Profiler (whole transcriptome)	NanoString	N/A	Spatial profiling of CHLA-10 xenografts (data from Wrenn et al., 2023)
Commercial assay or kit	Intercept (TBS) Blocking Buffer (formerly Odyssey Blocking Buffer)	LI-COR Biosciences	N/A	Membrane blocking and secondary-antibody diluent for western blots
Commercial assay or kit	4–15% Mini-PROTEAN TGX gels / Mini-PROTEAN Tetra system	Bio-Rad	N/A	SDS–PAGE for western blots
Chemical compound, drug	Puromycin	Gibco/Thermo Fisher Scientific	Cat# A1113802	1.5 µg/ml for selection of transduced cells
Chemical compound, drug	Polyethylenimine (PEI)	Kyfora Bio	Cat# 24765-100	Transfection reagent for lentivirus production
Chemical compound, drug	Sodium butyrate	Sigma	Cat# B5887-1G	Added to packaging cells to enhance viral production
Software, algorithm	Cell Ranger ARC	10x Genomics	RRID:SCR_023897	Multiome read demultiplexing and processing
Software, algorithm	Cell Ranger	10x Genomics	RRID:SCR_017344	scRNA-seq processing of primary patient tumors
Software, algorithm	ArchR	Granja et al., 2021	RRID:SCR_020982	Single-cell multiome/chromatin accessibility analysis
Software, algorithm	Signac	Stuart et al., 2021	RRID:SCR_021158	scATAC/weighted-nearest-neighbor analysis
Software, algorithm	Seurat	Stuart et al., 2019	RRID:SCR_016341	Single-cell integration, clustering, AddModuleScore
Software, algorithm	Souporcell	Heaton et al., 2020; Heaton, 2026	https://github.com/wheaton5/souporcell	Genotype-based demultiplexing of pooled cell lines
Software, algorithm	HOMER	Heinz et al., 2010	RRID:SCR_010881	Motif enrichment (findMotifsGenome.pl, annotatePeaks.pl)
Software, algorithm	chromVAR	Schep et al.	RRID:SCR_026570	Single-cell TF motif deviation scores
Software, algorithm	JASPAR2020	JASPAR	RRID:SCR_003030	Motif database for EWS::FLI1 deviation analysis
Software, algorithm	cisBP	Weirauch et al., 2014	http://cisbp.ccbr.utoronto.ca	Transcription factor motif database
Software, algorithm	ComplexHeatmap	Gu et al.	RRID:SCR_017270	Heatmap visualization
Software, algorithm	clusterProfiler	Yu et al.	RRID:SCR_016884	Gene set/over-representation enrichment (enricher)
Software, algorithm	MSigDB	Subramanian et al., 2005	RRID:SCR_016863	Hallmark and Gene Ontology gene-set references
Software, algorithm	decoupleR	Badia-I-Mompel et al., 2022; Badia-i-Mompel et al., 2026	https://saezlab.github.io/decoupleR	Pathway activity inference
Software, algorithm	CellBender	Broad Institute; Broad Institute, 2026	https://github.com/broadinstitute/CellBender	Ambient RNA removal for patient scRNA-seq
Software, algorithm	cellSignalAnalysis	Young et al., 2021; Young, 2022	https://github.com/constantAmateur/cellSignalAnalysis	Bulk RNA-seq deconvolution against single-cell reference
Software, algorithm	IsoSeq3 (lima, tag, refine, correct, dedup, pbmm2)	PacificBiosciences, 2026a	https://github.com/PacificBiosciences/IsoSeq	Long-read CCS processing pipeline
Software, algorithm	pbfusion	PacificBiosciences, 2026b	https://github.com/PacificBiosciences/pbfusion	Long-read fusion-transcript detection
Software, algorithm	STAR	Dobin et al., 2013	RRID:SCR_004463	Bulk RNA-seq alignment
Software, algorithm	piperna	This paper/Furlan lab	https://github.com/furlan-lab/piperna	Bulk RNA-seq alignment/quantification workflow
Software, algorithm	oligo (Bioconductor)	Bioconductor	RRID:SCR_015729	Microarray normalization (with pd.huex.1.0.st.v2 annotation)
Software, algorithm	R	R Foundation	RRID:SCR_001905	Statistical computing environment
Software, algorithm	Analysis code (EwS_multiome)	This paper/Furlan lab (Waltner, 2026)	https://github.com/furlan-lab/EwS_multiome	All analysis code and processed data
Other	NovaSeq 6000 sequencer	Illumina	N/A	Short-read sequencing (~25K ATAC, ~20K RNA reads/cell)
Other	Sequel IIe sequencer	Pacific Biosciences	N/A	Long-read sequencing of enriched EWS::FLI1 cDNA
Other	Odyssey imaging system	LI-COR Biosciences	N/A	Western blot scanning

### Sex as a biological variable

Cell lines and patient samples from both male and female patients were included; sex was not analyzed as a biological variable.

### Cell lines and PDX

The EwS cell lines A673, SKNMC, CHLA10, CHLA9, TC32, A4573, RDES, and TC71 were obtained from ATCC and COG (https://www.childrensoncologygroup.org/) cell lines repositories. The osteosarcoma cell line U2OS and rhabdomyosarcoma cell line RD were also obtained from ATCC. 5046L and 240L MSCs were obtained from Dr. Darwin Prockop. H7 MSCs were generously gifted from Dr. Alejandro Sweet-Cordero. PDX305 was generated from a recurrent metastatic tumor, as previously described (Wrenn et al., 2023). A673, TC32, A4573, TC71, and SKNMC cell lines were maintained in RPMI 1640 media (Gibco) supplemented with 10% FBS (Atlas Biologicals) and 2 mM L-glutamine (Life Technologies). RDES was maintained in RPMI 1640 media supplemented with 15% FBS. Isogenic cell lines, CHLA10 and CHLA9, were maintained in IMDM media (Fisher) supplemented with 20% FBS, 2 mmol/L-glutamine, and 1X Insulin-Transferrin-Selenium (Gibco). PDX305 was maintained in RPMI 1640 media supplemented with 10% FBS, 2 mmol/L-glutamine, and 1% Antimycotic-Antibiotic (Gibco). U2OS was maintained in McCoy’s 5A media supplemented with 10% FBS and 2 mmol/L-glutamine, and RD was maintained in DMEM with 10% FBS. All hMSC lines were maintained in MEM-alpha media supplemented with 20% FBS, 2 mmol/L-glutamine, and 1% Antimycotic-Antibiotic. Cells were cultured at 37°C with 5% CO_2_. Cells were all confirmed to be mycoplasma free, and identities subject to STR-confirmation every 6 months.

### Lentivirus production and genetic knockdown

pLKO.1 shNS (SHC002) and shFLI1 (TRCN0000005322) were used for FLI1 knockdown multiome experiments (Sigma). Knockdown plasmids were co-transfected with pCD/NL-BH*DDD (addgene, #17531) and pMD2.G (addgene, #12259) plasmids into 293FT packaging cells using PEI. 24 hr post-transfection, sodium butyrate was added to plates for 6 hr. Viral supernatant was collected 48 hr after transfection, filtered through 40 µM filters, and added to the cells for 24 hr. EwS cells were selected by supplementing the media with 1.5 µg/ml puromycin (Gibco) for 48 hr before collection at 96 hr.

### TGF-β treatment prior to single-cell sequencing

TC71, A673, and CHLA10 cells were plated at 500,000 viable cells per 10 cm plate. 48 hr later, recombinant human TGF-β1 (R&D 7754-BH-005, final dose 10 ng/ml in sterile 4 mM HCl and 0.1% BSA) or vehicle control was added. 24 hr later, cells were trypsinized and >1 × 10^6^ viable cells were subjected to single-cell library preparation and sequencing (details below).

### Western blots

Cells were washed with ice cold PBS, scraped and centrifuged, then lysed with RIPA buffer (Fisher Scientific) supplemented with protease and phosphatase inhibitors (Sigma) and sonicated (ten 30-s cycles). For TGF-β treated samples, recombinant TGF-β1 (10 ng/ml) or vehicle control (4 mM HCl, 0.1% BSA) were included in the media for either 30 min or 24 hr before lysis. Western blots were performed using the Bio-Rad Mini-PROTEAN Tetra System using 40 µg of lysate and 4–15% Mini-PROTEAN TGX polyacrylamide gels. Following transfer, nitrocellulose membranes were blocked in Intercept (TBS) Blocking Buffer for 1 hr. Membranes were washed and incubated rotating overnight at 4°C with primary antibodies (FLI1 Abcam ab133485, 1:1000; FOSL2 CST #19967, HAND2 R&D AF3876, RUNX3 CST #13089, TEAD1 CST #12292, pSMAD2 S465/467 CST #3108, SMAD2 CST #3103, GAPDH Invitrogen AM4300, Vinculin CST #13901). Membranes were then washed four times in TBST for 5 min each and incubated with secondary antibodies (LI-COR IRDye 680RD or 800CW; 1:10,000; goat anti-mouse 926-32210, goat anti-rabbit 926-68071, and donkey anti-goat 926-68074) for 1 hr. After four additional 5-min TBST washes, membranes were scanned on a LI-COR Odyssey scanner.

### Digital spatial profiling

Q3 normalized gene counts from GeoMx Digital Spatial Profiling (NanoString) of EwS cell line xenograft tumors formed by CHLA10 injection were obtained from previous work (Wrenn et al., 2023). This processed data was used to query fusion regulated module gene sets with respect to tumor microenvironment and injection site. Gene set values for each ROI were calculated by converting the DSP Q3 counts matrix into a Seurat object and using the AddModuleScore function.

### Single-cell analysis

#### Cell and library prep

One million EwS cells were resuspended in PBS containing 2% BSA, and nuclei were prepared according to manufacturer’s specifications. Cell lines in triplicate were pooled 1:1:1 at 1000 cells/µl and libraries were generated using the Chromium Next GEM Single-Cell Multiome ATAC + Gene Expression Kit following the manufacturer’s protocol (CG000338 Rev C). The resulting libraries were sequenced using a NovaSeq 6000 targeting a depth of 25K reads per cell (ATAC libraries) and 20K reads per cell (RNA libraries).

#### Single-cell multiome data analysis

Sequencing reads were demultiplexed and processed using the cellranger ARC pipeline. Cells with poor quality ATAC fragments and multiplets were filtered using ArchR’s standard workflow. Cells passing the following were kept for later analysis: percent mitochondrial RNA reads <15%; 3 < log_10_(RNA/UMI counts) ≤ 4.5. Souporcell (Heaton et al., 2020) was used to identify cell lines and remove additional multiplets. Top RNA markers from scRNA data were compared to previously hashed CITEseq profiles (Wrenn et al., 2023) to match genotype to cell line (GSE236289: GSM7527501, GSM7527502, and GSM7527503). ArchR (Granja et al., 2021), Signac (Stuart et al., 2021), and Seurat (Stuart et al., 2019) were used for downstream analysis. Dimensionality reduction was performed using addIterativeLSI() for both tile and gene expression matrices. An embedding was created by combineDims() and then addUMAP() with the multiomic reduction. Group coverages and peak calls were made using cell line identities. The motif matrix was built using addMotifDeviations(), addBdgPeaks(), and addDeviationsMatrix().

Average gene expression and gene activity scores were computed using addModuleScore(). EWS::FLI1 deviations were computed using the JASPAR2020 motif database. TF analyses used the cisBP database (Weirauch et al., 2014). Highly utilized TFs were inferred using correlateMatrices(). All heatmaps were made with ComplexHeatmap. Marker peaks and genes were calculated using getMarkerFeatures() and getMarkersHeatmap(). Cis-regulatory elements were identified using addPeak2GeneLinks(). Universal gene set enrichment analysis was performed using clusterProfiler using the function enricher(), with MSigDB (Subramanian et al., 2005) Hallmark and gene ontology references. Motif enrichment analysis on kmean peak data was performed using HOMER. The most enriched motifs were identified using findMotifsGenome.pl, and motif enrichment was computed and visualized using annotatePeaks.pl. EWS::ETS binding sites were sourced from previously published data (Orth et al., 2022), and analyzed using HOMER and chromVAR with the same workflow as above.

Cells treated with TGF-β agonist and captured for multiomic sequencing were demultiplexed and processed using the same workflow as above. QC metrics used were identical to those above. A similar method of identifying each cell line using genetic demultiplexing was employed. Signac was used to perform dimensionality reduction using weighted nearest neighbors. Motif matrices were constructed using RunChromVAR(), using cisBP (Weirauch et al., 2014) as a database.

#### EWS::FLI1 long read sequencing

Starting material was 10x Genomics-generated full-length barcoded cDNA, leftover from what was used to create the Illumina-compatible libraries in the multiome downstream steps. Because 10x Genomics single-cell cDNA preparations typically contain a template switch oligo (TSO) priming artifact (i.e. TSO sequence at both ends of the cDNA molecule) instead of the correct structure, TSO artifact depletion was performed by using biotin-labeled primers for the streptavidin/biotin magnetic selection of molecules with the correct structure, as previously described by Al’Khafaji et al., 2024. To capture the gene transcripts encoding the EWS::FLI1 fusions, an enrichment was performed using xGen Hybridization and Wash v2 kit magnetic beads and reagents (IDT #10010351), as described in the manufacturer’s protocol, in addition to using 500 ng of cDNA starting material and using biotinylated capture probes designed with high specificity, tiling along exons 5, 6, and 7 of the *EWSR1* transcript, and exon 9 of the *FLI1* transcript. Following hybridization and magnetic capture of EWS::FLI1 molecules, an on-bead PCR was performed to amplify captured molecules, using standard 10x Genomic cDNA amplification protocol (described in 10x Genomics document #CG000315_Rev_B, Step 2.2) with the exception of a number of PCR cycles of 16 and an extension time at 72°C of 3 min. Following this, 500 ng of captured cDNA was DNA-repaired, A-tailed, SMRTbell adaptor-ligated, nuclease-treated, and SMRTbell-cleanup purified using the PacBio SMRTbell Prep kit 3.0 (PacBio #102-141-700) as per the manufacturer’s protocol. Libraries were sequenced on a PacBio Sequel IIe with 2 hr pre-extension time and 30 hr movie time.

Long-read computational processing pipeline consisted of retrieving error-corrected circular consensus sequence (CCS) BAM files generated by the on-board PacBio Sequel IIe (Pacific Biosciences of California, Inc). CCS data were then processed by the programs in the IsoSeq3 suite of computational tools: (1) *Lima* to remove sequencing primers, (2) *Tag* to extract UMI and CB sequences, (3) *Refine* to remove polyA sequences, (4) *Correct* to error-correct CBs, (5) *Dedup* to remove PCR duplicates, and (6) *Pbmm2* to align reads. All the IsoSeq3 pipeline steps were used with default settings. Mapped BAMs were then used for pbfusion to detect fusions. Barcodes from long read molecules were extracted from mapped BAMs and merged with data of pbfusion.breakpoints.groups.bed files to count fusion transcripts in single cells.

### External data

The 78 direct EwS TGs were sourced from a previously published dataset (Aynaud et al., 2020). Bulk RNA-seq patient tumor samples were sourced from dbGaP under accession numbers PRJNA253759 and PRJNA261990. FASTQ files were aligned to the reference genome (GRCh38) and using the piperna (https://github.com/furlan-lab/piperna, furlan-lab, 2022) workflow which uses STAR (Dobin et al., 2013) aligner and generates a count matrix using the Bioconductor summarize overlap function (https://www.bioconductor.org) with its default settings. Microarray data were accessed from GSE63157 (Volchenboum et al., 2015). RNA probes were annotated using pd.huex.1.0.st.v2, and normalized using oligo.

### scRNA-seq patient tumors

Patient tumor biopsies were sourced and sequenced at St. Jude Children’s Research Hospital (Hawkins et al., 2026). 10X reads were demultiplexed and processed using the cellranger pipeline. Ambient RNA was filtered out using cellbender. Cells with poor RNA measurements were filtered using the following thresholds: percent mitochondrial reads <15% and 3 < log_10_(RNA counts) < 5.25. Dimensionality reduction and clustering were performed using standard workflow. Surrounding tissue cells were identified by using FindAllMarkers on Seurat clusters. Tumor enrichment was validated by choosing cells that express *CD99*, *NR0B1*, and EwS TGs. Identified tumor cells underwent normalization and dimensionality reduction with the same workflow. Average gene set scores were calculated using AddModuleScore().

### Deconvolution

Bulk RNA-seq patient data were deconvoluted using cellSignalAnalysis (https://github.com/constantAmateur/cellSignalAnalysis, Young, 2022). EwS subgroups 1–3 were used as transcriptomic references.

### Statistics

All statistical analyses were conducted using R or Python, with specific packages indicated in relevant sections of the Methods. Statistical significance was assessed using two-sided tests unless otherwise specified. For comparisons of gene set scores, motif deviations, or expression levels, Wilcoxon rank-sum tests were used. Correction for multiple hypothesis testing was performed using the Benjamini–Hochberg method where applicable. Gene set enrichment analyses were conducted using the clusterProfiler package with MSigDB Hallmark and Gene Ontology annotations. For scRNA-seq and multiome analyses, differential expression and peak calling were performed using Seurat, ArchR, or Signac (Stuart et al., 2021), and marker features were identified with adjusted p-values <0.05 considered significant. Event-free survival was compared between tumors above and below the 85th percentile of module score or FOSL2 expression using a Cox proportional hazards model. Correlations were assessed by Pearson correlation.

### Study approval

All work was approved by the Fred Hutchinson Cancer Center Institutional Review Board—Tumor Heterogeneity and Metastasis in Ewing Sarcoma—FHIRB0010627. No human participants were recruited for this study. All experiments used established cell lines, and all patient data were de-identified and previously published; informed consent was obtained by the original investigators, and no additional consent was required.

## Data Availability

All code and processed data is available on https://github.com/furlan-lab/EwS_multiome (copy archived at Waltner, 2026). Data has also been deposited on GEO with accession GSE332630, including raw sequence. Single-cell RNA sequencing from primary samples is available at https://scpca.alexslemonade.org. The following dataset was generated: FurlanSN 2026Multimodal single-cell analyses reveal distinct fusion-regulated transcriptional programs in Ewing sarcomaNCBI Gene Expression OmnibusGSE33263010.7554/eLife.109124PMC1375427843012345

## Additional files

MDAR checklist

## References

[bib1] Adane B, Alexe G, Seong BKA, Lu D, Hwang EE, Hnisz D, Lareau CA, Ross L, Lin S, Dela Cruz FS, Richardson M, Weintraub AS, Wang S, Iniguez AB, Dharia NV, Conway AS, Robichaud AL, Tanenbaum B, Krill-Burger JM, Vazquez F, Schenone M, Berman JN, Kung AL, Carr SA, Aryee MJ, Young RA, Crompton BD, Stegmaier K (2021). STAG2 loss rewires oncogenic and developmental programs to promote metastasis in Ewing sarcoma. Cancer Cell.

[bib2] Al’Khafaji AM, Smith JT, Garimella KV, Babadi M, Popic V, Sade-Feldman M, Gatzen M, Sarkizova S, Schwartz MA, Blaum EM, Day A, Costello M, Bowers T, Gabriel S, Banks E, Philippakis AA, Boland GM, Blainey PC, Hacohen N (2024). High-throughput RNA isoform sequencing using programmed cDNA concatenation. Nature Biotechnology.

[bib3] Apfelbaum AA, Wrenn ED, Lawlor ER (2022a). The importance of fusion protein activity in Ewing sarcoma and the cell intrinsic and extrinsic factors that regulate it: A review. Frontiers in Oncology.

[bib4] Apfelbaum AA, Wu F, Hawkins AG, Magnuson B, Jiménez JA, Taylor SD, Wrenn ED, Waltner O, Pfaltzgraff ER, Song JY, Hall C, Wellik DM, Ljungman M, Furlan SN, Ryan RJH, Sarthy JF, Lawlor ER (2022b). EWS::FLI1 and HOXD13 control tumor cell plasticity in ewing sarcoma. Clinical Cancer Research.

[bib5] Aynaud MM, Mirabeau O, Gruel N, Grossetête S, Boeva V, Durand S, Surdez D, Saulnier O, Zaïdi S, Gribkova S, Fouché A, Kairov U, Raynal V, Tirode F, Grünewald TGP, Bohec M, Baulande S, Janoueix-Lerosey I, Vert JP, Barillot E, Delattre O, Zinovyev A (2020). Transcriptional programs define intratumoral heterogeneity of ewing sarcoma at single-cell resolution. Cell Reports.

[bib6] Badia-I-Mompel P, Vélez Santiago J, Braunger J, Geiss C, Dimitrov D, Müller-Dott S, Taus P, Dugourd A, Holland CH, Ramirez Flores RO, Saez-Rodriguez J (2022). decoupleR: ensemble of computational methods to infer biological activities from omics data. Bioinformatics Advances.

[bib7] Badia-i-Mompel P, Vélez-Santiago J, Braunger J, Geiss C, Dimitrov D, Müller-Dott S, Taus P, Dugourd A, Holland CH, Ramirez Flores RO, Saez-Rodriguez J (2026). GitHub.

[bib8] Bilke S, Schwentner R, Yang F, Kauer M, Jug G, Walker RL, Davis S, Zhu YJ, Pineda M, Meltzer PS, Kovar H (2013). Oncogenic ETS fusions deregulate E2F3 target genes in Ewing sarcoma and prostate cancer. Genome Research.

[bib9] Bledsoe KL, McGee-Lawrence ME, Camilleri ET, Wang X, Riester SM, van Wijnen AJ, Oliveira AM, Westendorf JJ (2014). RUNX3 facilitates growth of Ewing sarcoma cells. Journal of Cellular Physiology.

[bib10] Boeva V, Louis-Brennetot C, Peltier A, Durand S, Pierre-Eugène C, Raynal V, Etchevers HC, Thomas S, Lermine A, Daudigeos-Dubus E, Geoerger B, Orth MF, Grünewald TGP, Diaz E, Ducos B, Surdez D, Carcaboso AM, Medvedeva I, Deller T, Combaret V, Lapouble E, Pierron G, Grossetête-Lalami S, Baulande S, Schleiermacher G, Barillot E, Rohrer H, Delattre O, Janoueix-Lerosey I (2017). Heterogeneity of neuroblastoma cell identity defined by transcriptional circuitries. Nature Genetics.

[bib11] Boulay G, Sandoval GJ, Riggi N, Iyer S, Buisson R, Naigles B, Awad ME, Rengarajan S, Volorio A, McBride MJ, Broye LC, Zou L, Stamenkovic I, Kadoch C, Rivera MN (2017). Cancer-specific retargeting of BAF complexes by a prion-like domain. Cell.

[bib12] (2026). GitHub.

[bib13] Brohl AS, Solomon DA, Chang W, Wang J, Song Y, Sindiri S, Patidar R, Hurd L, Chen L, Shern JF, Liao H, Wen X, Gerard J, Kim JS, Lopez Guerrero JA, Machado I, Wai DH, Picci P, Triche T, Horvai AE, Miettinen M, Wei JS, Catchpool D, Llombart-Bosch A, Waldman T, Khan J (2014). The genomic landscape of the Ewing Sarcoma family of tumors reveals recurrent STAG2 mutation. PLOS Genetics.

[bib14] Chaturvedi A, Hoffman LM, Welm AL, Lessnick SL, Beckerle MC (2012). The EWS/FLI oncogene drives changes in cellular morphology, adhesion, and migration in ewing sarcoma. Genes & Cancer.

[bib15] Chaturvedi A, Hoffman LM, Jensen CC, Lin YC, Grossmann AH, Randall RL, Lessnick SL, Welm AL, Beckerle MC (2014). Molecular dissection of the mechanism by which EWS/FLI expression compromises actin cytoskeletal integrity and cell adhesion in Ewing sarcoma. Molecular Biology of the Cell.

[bib16] Crompton BD, Stewart C, Taylor-Weiner A, Alexe G, Kurek KC, Calicchio ML, Kiezun A, Carter SL, Shukla SA, Mehta SS, Thorner AR, de Torres C, Lavarino C, Suñol M, McKenna A, Sivachenko A, Cibulskis K, Lawrence MS, Stojanov P, Rosenberg M, Ambrogio L, Auclair D, Seepo S, Blumenstiel B, DeFelice M, Imaz-Rosshandler I, Schwarz-Cruz Y Celis A, Rivera MN, Rodriguez-Galindo C, Fleming MD, Golub TR, Getz G, Mora J, Stegmaier K (2014). The genomic landscape of pediatric Ewing sarcoma. Cancer Discovery.

[bib17] Croushore EE, Stipp CS, Gordon DJ (2024). EWS-FLI1 and activator protein-1 (AP-1) reciprocally regulate extracellular-matrix proteins in ewing sarcoma cells. International Journal of Molecular Sciences.

[bib18] Davies A, Zoubeidi A, Beltran H, Selth LA (2023). The transcriptional and epigenetic landscape of cancer cell lineage plasticity. Cancer Discovery.

[bib19] Dobin A, Davis CA, Schlesinger F, Drenkow J, Zaleski C, Jha S, Batut P, Chaisson M, Gingeras TR (2013). STAR: ultrafast universal RNA-seq aligner. Bioinformatics.

[bib20] Faustino A, Couto JP, Pópulo H, Rocha AS, Pardal F, Cameselle-Teijeiro JM, Lopes JM, Sobrinho-Simões M, Soares P (2012). mTOR pathway overactivation in BRAF mutated papillary thyroid carcinoma. The Journal of Clinical Endocrinology and Metabolism.

[bib21] Franzetti GA, Laud-Duval K, van der Ent W, Brisac A, Irondelle M, Aubert S, Dirksen U, Bouvier C, de Pinieux G, Snaar-Jagalska E, Chavrier P, Delattre O (2017). Cell-to-cell heterogeneity of EWSR1-FLI1 activity determines proliferation/migration choices in Ewing sarcoma cells. Oncogene.

[bib22] (2022). Github.

[bib23] García-García L, Fernández-Tabanera E, Cervera ST, Melero-Fernández de Mera RM, Josa S, González-González L, Rodríguez-Martín C, Grünewald TGP, Alonso J (2021). The transcription factor *FEZF1*, a Direct Target of *EWSR1-FLI1* in Ewing sarcoma cells, regulates the expression of neural-specific genes. Cancers.

[bib24] Gavish A, Tyler M, Greenwald AC, Hoefflin R, Simkin D, Tschernichovsky R, Galili Darnell N, Somech E, Barbolin C, Antman T, Kovarsky D, Barrett T, Gonzalez Castro LN, Halder D, Chanoch-Myers R, Laffy J, Mints M, Wider A, Tal R, Spitzer A, Hara T, Raitses-Gurevich M, Stossel C, Golan T, Tirosh A, Suvà ML, Puram SV, Tirosh I (2023). Hallmarks of transcriptional intratumour heterogeneity across a thousand tumours. Nature.

[bib25] Goodspeed A, Bodlak A, Duffy AB, Nelson-Taylor S, Oike N, Porfilio T, Shirai R, Walker D, Treece A, Black J, Donaldson N, Cost C, Garrington T, Greffe B, Luna-Fineman S, Demedis J, Lake J, Danis E, Verneris MR, Adams DL, Hayashi M (2025). Single-Cell RNA sequencing of ewing sarcoma tumors demonstrates transcriptional heterogeneity and clonal evolution. Clinical Cancer Research.

[bib26] Granja JM, Corces MR, Pierce SE, Bagdatli ST, Choudhry H, Chang HY, Greenleaf WJ (2021). ArchR is a scalable software package for integrative single-cell chromatin accessibility analysis. Nature Genetics.

[bib27] Grünewald TGP, Cidre-Aranaz F, Surdez D, Tomazou EM, de Álava E, Kovar H, Sorensen PH, Delattre O, Dirksen U (2018). Ewing sarcoma. Nature Reviews. Disease Primers.

[bib28] Hanahan D (2022). Hallmarks of cancer: New dimensions. Cancer Discovery.

[bib29] Hawkins AG, Pedersen EA, Treichel S, Temprine K, Sperring C, Read JA, Magnuson B, Chugh R, Lawlor ER (2020). Wnt/β-catenin-activated Ewing sarcoma cells promote the angiogenic switch. JCI Insight.

[bib30] Hawkins AG, Shapiro JA, Spielman SJ, Mejia DS, Venkatesh Prasad D, Ichihara N, Yakovets A, Gottlieb AM, Wheeler KG, Bethell CJ, Foltz SM, O’Malley J, Greene CS, Taroni JN (2026). The single-cell pediatric cancer atlas: data portal and open-source tools for single-cell transcriptomics of pediatric tumors. Cell Genomics.

[bib31] Heaton H, Talman AM, Knights A, Imaz M, Gaffney DJ, Durbin R, Hemberg M, Lawniczak MKN (2020). Souporcell: robust clustering of single-cell RNA-seq data by genotype without reference genotypes. Nature Methods.

[bib32] Heaton H (2026). GitHub.

[bib33] Heinz S, Benner C, Spann N, Bertolino E, Lin YC, Laslo P, Cheng JX, Murre C, Singh H, Glass CK (2010). Simple combinations of lineage-determining transcription factors prime cis-regulatory elements required for macrophage and B cell identities. Molecular Cell.

[bib34] Johnson KM, Taslim C, Saund RS, Lessnick SL (2017). Identification of two types of GGAA-microsatellites and their roles in EWS/FLI binding and gene regulation in Ewing sarcoma. PLOS ONE.

[bib35] Kakkar A, Majumdar A, Kumar A, Tripathi M, Pathak P, Sharma MC, Suri V, Tandon V, Chandra SP, Sarkar C (2016). Alterations in BRAF gene, and enhanced mTOR and MAPK signaling in dysembryoplastic neuroepithelial tumors (DNTs). Epilepsy Research.

[bib36] Katschnig AM, Kauer MO, Schwentner R, Tomazou EM, Mutz CN, Linder M, Sibilia M, Alonso J, Aryee DNT, Kovar H (2017). EWS-FLI1 perturbs MRTFB/YAP-1/TEAD target gene regulation inhibiting cytoskeletal autoregulatory feedback in Ewing sarcoma. Oncogene.

[bib37] Kim S, Denny CT, Wisdom R (2006). Cooperative DNA binding with AP-1 proteins is required for transformation by EWS-Ets fusion proteins. Molecular and Cellular Biology.

[bib38] Kinsey M, Smith R, Lessnick SL (2006). NR0B1 is required for the oncogenic phenotype mediated by EWS/FLI in Ewing’s sarcoma. Molecular Cancer Research.

[bib39] Kumar RR, Agarwal N, Shree A, Gorain JK, Rahul E, Ganguly S, Bakhshi S, Sharma U (2025). Decoding the immune landscape in Ewing sarcoma pathogenesis: The role of tumor infiltrating immune cells and immune milieu. Journal of Bone Oncology.

[bib40] Lin PP, Wang Y, Lozano G (2011). Mesenchymal stem cells and the origin of Ewing’s Sarcoma. Sarcoma.

[bib41] Lin L, Huang M, Shi X, Mayakonda A, Hu K, Jiang YY, Guo X, Chen L, Pang B, Doan N, Said JW, Xie J, Gery S, Cheng X, Lin Z, Li J, Berman BP, Yin D, Lin DC, Koeffler HP (2019). Super-enhancer-associated MEIS1 promotes transcriptional dysregulation in Ewing sarcoma in co-operation with EWS-FLI1. Nucleic Acids Research.

[bib42] Lu DY, Ellegast JM, Ross KN, Malone CF, Lin S, Mabe NW, Dharia NV, Meyer A, Conway A, Su AH, Selich-Anderson J, Taslim C, Byrum AK, Seong BKA, Adane B, Gray NS, Rivera MN, Lessnick SL, Stegmaier K (2023). The ETS transcription factor ETV6 constrains the transcriptional activity of EWS-FLI to promote Ewing sarcoma. Nature Cell Biology.

[bib43] Marques Howarth M, Simpson D, Ngok SP, Nieves B, Chen R, Siprashvili Z, Vaka D, Breese MR, Crompton BD, Alexe G, Hawkins DS, Jacobson D, Brunner AL, West R, Mora J, Stegmaier K, Khavari P, Sweet-Cordero EA (2014). Long noncoding RNA EWSAT1-mediated gene repression facilitates Ewing sarcoma oncogenesis. The Journal of Clinical Investigation.

[bib44] Marusyk A, Almendro V, Polyak K (2012). Intra-tumour heterogeneity: a looking glass for cancer?. Nature Reviews Cancer.

[bib45] Miller HE, Gorthi A, Bassani N, Lawrence LA, Iskra BS, Bishop AJR (2020). Reconstruction of ewing sarcoma developmental context from mass-scale transcriptomics reveals characteristics of EWSR1-FLI1 permissibility. Cancers.

[bib46] Minas TZ, Surdez D, Javaheri T, Tanaka M, Howarth M, Kang H-J, Han J, Han Z-Y, Sax B, Kream BE, Hong S-H, Çelik H, Tirode F, Tuckermann J, Toretsky JA, Kenner L, Kovar H, Lee S, Sweet-Cordero EA, Nakamura T, Moriggl R, Delattre O, Üren A (2017). Combined experience of six independent laboratories attempting to create an Ewing sarcoma mouse model. Oncotarget.

[bib47] Orth MF, Surdez D, Faehling T, Ehlers AC, Marchetto A, Grossetête S, Volckmann R, Zwijnenburg DA, Gerke JS, Zaidi S, Alonso J, Sastre A, Baulande S, Sill M, Cidre-Aranaz F, Ohmura S, Kirchner T, Hauck SM, Reischl E, Gymrek M, Pfister SM, Strauch K, Koster J, Delattre O, Grünewald TGP (2022). Systematic multi-omics cell line profiling uncovers principles of Ewing sarcoma fusion oncogene-mediated gene regulation. Cell Reports.

[bib48] (2026a). GitHub.

[bib49] (2026b). GitHub.

[bib50] Pedersen EA, Menon R, Bailey KM, Thomas DG, Van Noord RA, Tran J, Wang H, Qu PP, Hoering A, Fearon ER, Chugh R, Lawlor ER (2016). Activation of Wnt/β-catenin in ewing sarcoma cells antagonizes EWS/ETS function and promotes phenotypic transition to more metastatic cell states. Cancer Research.

[bib51] Prabowo AS, Iyer AM, Veersema TJ, Anink JJ, Schouten-van Meeteren AYN, Spliet WGM, van Rijen PC, Ferrier CH, Capper D, Thom M, Aronica E (2014). BRAF V600E mutation is associated with mTOR signaling activation in glioneuronal tumors. Brain Pathology.

[bib52] Riggi N, Suva ML, Stamenkovic I (2009). Ewing’s sarcoma origin: from duel to duality. Expert Review of Anticancer Therapy.

[bib53] Riggi N, Knoechel B, Gillespie SM, Rheinbay E, Boulay G, Suvà ML, Rossetti NE, Boonseng WE, Oksuz O, Cook EB, Formey A, Patel A, Gymrek M, Thapar V, Deshpande V, Ting DT, Hornicek FJ, Nielsen GP, Stamenkovic I, Aryee MJ, Bernstein BE, Rivera MN (2014). EWS-FLI1 utilizes divergent chromatin remodeling mechanisms to directly activate or repress enhancer elements in Ewing sarcoma. Cancer Cell.

[bib54] Riggi N, Suvà ML, Stamenkovic I (2021). Ewing’s Sarcoma. New England Journal of Medicine.

[bib55] Sanalkumar R, Dong R, Lee L, Xing Y-H, Iyer S, Letovanec I, La Rosa S, Finzi G, Musolino E, Papait R, Chebib I, Nielsen GP, Renella R, Cote GM, Choy E, Aryee M, Stegmaier K, Stamenkovic I, Rivera MN, Riggi N (2023). Highly connected 3D chromatin networks established by an oncogenic fusion protein shape tumor cell identity. Science Advances.

[bib56] Sankar S, Bell R, Stephens B, Zhuo R, Sharma S, Bearss DJ, Lessnick SL (2013). Mechanism and relevance of EWS/FLI-mediated transcriptional repression in Ewing sarcoma. Oncogene.

[bib57] Sheffield NC, Pierron G, Klughammer J, Datlinger P, Schönegger A, Schuster M, Hadler J, Surdez D, Guillemot D, Lapouble E, Freneaux P, Champigneulle J, Bouvier R, Walder D, Ambros IM, Hutter C, Sorz E, Amaral AT, de Álava E, Schallmoser K, Strunk D, Rinner B, Liegl-Atzwanger B, Huppertz B, Leithner A, de Pinieux G, Terrier P, Laurence V, Michon J, Ladenstein R, Holter W, Windhager R, Dirksen U, Ambros PF, Delattre O, Kovar H, Bock C, Tomazou EM (2017). DNA methylation heterogeneity defines a disease spectrum in Ewing sarcoma. Nature Medicine.

[bib58] Shi X, Zheng Y, Jiang L, Zhou B, Yang W, Li L, Ding L, Huang M, Gery S, Lin DC, Koeffler HP (2020). EWS-FLI1 regulates and cooperates with core regulatory circuitry in Ewing sarcoma. Nucleic Acids Research.

[bib59] Smith R, Owen LA, Trem DJ, Wong JS, Whangbo JS, Golub TR, Lessnick SL (2006). Expression profiling of EWS/FLI identifies NKX2.2 as a critical target gene in Ewing’s sarcoma. Cancer Cell.

[bib60] Stuart T, Butler A, Hoffman P, Hafemeister C, Papalexi E, Mauck WM, Hao Y, Stoeckius M, Smibert P, Satija R (2019). Comprehensive integration of single-cell data. Cell.

[bib61] Stuart T, Srivastava A, Madad S, Lareau CA, Satija R (2021). Single-cell chromatin state analysis with Signac. Nature Methods.

[bib62] Subramanian A, Tamayo P, Mootha VK, Mukherjee S, Ebert BL, Gillette MA, Paulovich A, Pomeroy SL, Golub TR, Lander ES, Mesirov JP (2005). Gene set enrichment analysis: A knowledge-based approach for interpreting genome-wide expression profiles. PNAS.

[bib63] Surdez D, Zaidi S, Grossetête S, Laud-Duval K, Ferre AS, Mous L, Vourc’h T, Tirode F, Pierron G, Raynal V, Baulande S, Brunet E, Hill V, Delattre O (2021). STAG2 mutations alter CTCF-anchored loop extrusion, reduce cis-regulatory interactions and EWSR1-FLI1 activity in Ewing sarcoma. Cancer Cell.

[bib64] Tirode F, Laud-Duval K, Prieur A, Delorme B, Charbord P, Delattre O (2007). Mesenchymal stem cell features of Ewing tumors. Cancer Cell.

[bib65] Tirosh I, Izar B, Prakadan SM, Wadsworth MH, Treacy D, Trombetta JJ, Rotem A, Rodman C, Lian C, Murphy G, Fallahi-Sichani M, Dutton-Regester K, Lin JR, Cohen O, Shah P, Lu D, Genshaft AS, Hughes TK, Ziegler CGK, Kazer SW, Gaillard A, Kolb KE, Villani AC, Johannessen CM, Andreev AY, Van Allen EM, Bertagnolli M, Sorger PK, Sullivan RJ, Flaherty KT, Frederick DT, Jané-Valbuena J, Yoon CH, Rozenblatt-Rosen O, Shalek AK, Regev A, Garraway LA (2016). Dissecting the multicellular ecosystem of metastatic melanoma by single-cell RNA-seq. Science.

[bib66] Tomazou EM, Sheffield NC, Schmidl C, Schuster M, Schönegger A, Datlinger P, Kubicek S, Bock C, Kovar H (2015). Epigenome mapping reveals distinct modes of gene regulation and widespread enhancer reprogramming by the oncogenic fusion protein EWS-FLI1. Cell Reports.

[bib67] van Groningen T, Koster J, Valentijn LJ, Zwijnenburg DA, Akogul N, Hasselt NE, Broekmans M, Haneveld F, Nowakowska NE, Bras J, van Noesel CJM, Jongejan A, van Kampen AH, Koster L, Baas F, van Dijk-Kerkhoven L, Huizer-Smit M, Lecca MC, Chan A, Lakeman A, Molenaar P, Volckmann R, Westerhout EM, Hamdi M, van Sluis PG, Ebus ME, Molenaar JJ, Tytgat GA, Westerman BA, van Nes J, Versteeg R (2017). Neuroblastoma is composed of two super-enhancer-associated differentiation states. Nature Genetics.

[bib68] Visser LL, Bleijs M, Margaritis T, van de Wetering M, Holstege FCP, Clevers H (2023). Ewing sarcoma single-cell transcriptome analysis reveals functionally impaired antigen-presenting cells. Cancer Research Communications.

[bib69] Volchenboum SL, Andrade J, Huang L, Barkauskas DA, Krailo M, Womer RB, Ranft A, Potratz J, Dirksen U, Triche TJ, Lawlor ER (2015). Gene expression profiling of ewing sarcoma tumors reveals the prognostic importance of tumor-stromal interactions: A report from the children’s oncology group. The Journal of Pathology Clinical Research.

[bib70] Waltner O (2026). Software Heritage.

[bib71] Weirauch MT, Yang A, Albu M, Cote AG, Montenegro-Montero A, Drewe P, Najafabadi HS, Lambert SA, Mann I, Cook K, Zheng H, Goity A, van Bakel H, Lozano JC, Galli M, Lewsey MG, Huang E, Mukherjee T, Chen X, Reece-Hoyes JS, Govindarajan S, Shaulsky G, Walhout AJM, Bouget FY, Ratsch G, Larrondo LF, Ecker JR, Hughes TR (2014). Determination and inference of eukaryotic transcription factor sequence specificity. Cell.

[bib72] Wrenn ED, Apfelbaum AA, Rudzinski ER, Deng X, Jiang W, Sud S, Van Noord RA, Newman EA, Garcia NM, Miyaki A, Hoglund VJ, Bhise SS, Kanaan SB, Waltner OG, Furlan SN, Lawlor ER (2023). Cancer-associated fibroblast-like tumor cells remodel the ewing sarcoma tumor microenvironment. Clinical Cancer Research.

[bib73] Wrenn ED, Harris JC, Apfelbaum AA, Valenti JR, Lipson PA, Walter SI, Garcia NM, Miyaki A, Chen SC, Olson JM, Price JS, Bailey KM, Lawlor ER (2025). Autocrine TGFβ2 enforces a transcriptionally hybrid cell state in ewing sarcoma. bioRxiv.

[bib74] Young MD, Mitchell TJ, Custers L, Margaritis T, Morales-Rodriguez F, Kwakwa K, Khabirova E, Kildisiute G, Oliver TRW, de Krijger RR, van den Heuvel-Eibrink MM, Comitani F, Piapi A, Bugallo-Blanco E, Thevanesan C, Burke C, Prigmore E, Ambridge K, Roberts K, Braga FAV, Coorens THH, Del Valle I, Wilbrey-Clark A, Mamanova L, Stewart GD, Gnanapragasam VJ, Rampling D, Sebire N, Coleman N, Hook L, Warren A, Haniffa M, Kool M, Pfister SM, Achermann JC, He X, Barker RA, Shlien A, Bayraktar OA, Teichmann SA, Holstege FC, Meyer KB, Drost J, Straathof K, Behjati S (2021). Single cell derived mRNA signals across human kidney tumors. Nature Communications.

[bib75] Young MD (2022). Github.

[bib76] Zhang H, Liu CY, Zha ZY, Zhao B, Yao J, Zhao S, Xiong Y, Lei QY, Guan KL (2009). TEAD transcription factors mediate the function of TAZ in cell growth and epithelial-mesenchymal transition. The Journal of Biological Chemistry.

[bib77] Zoubek A, Dockhorn-Dworniczak B, Delattre O, Christiansen H, Niggli F, Gatterer-Menz I, Smith TL, Jürgens H, Gadner H, Kovar H (1996). Does expression of different EWS chimeric transcripts define clinically distinct risk groups of Ewing tumor patients?. Journal of Clinical Oncology.

