# Supplementary material for: Multimodal single-cell analyses reveal distinct fusion-regulated transcriptional programs in Ewing sarcoma
Source: eLife. 2026 Nov 11;14:RP109124. doi: 10.7554/eLife.109124 (PMC13754278; doi:10.7554/eLife.109124)
Supplement: Supplementary file 1 [file elife-109124-mdarchecklist1.pdf]

Materials Design Analysis Reporting (MDAR) Checklist for Authors

The MDAR framework establishes a minimum set of requirements in transparent reporting mainly applicable to studies in the life sciences.

eLife asks authors to **provide detailed information within their article** to facilitate the interpretation and replication of their work. Authors can also upload supporting materials to comply with relevant reporting guidelines for health-related research (see EQUATOR Network), life science research (see the BioSharing Information Resource), or animal research (see the ARRIVE Guidelines and the STRANGE Framework; for details, see *eLife*'s Journal Policies). Where applicable, authors should refer to any relevant reporting standards materials in this form.

For all that apply, please note **where in the article** the information is provided. Please note that we also collect information about data availability and ethics in the submission form.

Materials:

Newly created materials	Indicate where provided: section/figure legend	N/A
The manuscript includes a dedicated "materials availability statement" providing transparent disclosure about availability of newly created materials including details on how materials can be accessed and describing any restrictions on access.	Methods - 'Data and materials availability'; Key Resources Table. No new cell lines, animal models or plasmids were generated (PDX305 previously described, ref. 21; shRNA constructs commercial, Sigma). Custom EWS::FLI1 capture-probe sequences are given in the Key Resources Table.	

Antibodies	Indicate where provided: section/figure legend	N/A
For commercial reagents, provide supplier name, catalogue number and RRID, if available.	Key Resources Table: supplier, catalogue number, clone and RRID for all primary and LI-COR IRDye secondary antibodies; Methods - 'Western blots' (dilutions).	

DNA and RNA sequences	Indicate where provided: section/figure legend	N/A
Short novel DNA or RNA including primers, probes: Sequences should be included or deposited in a public repository.	Key Resources Table: full sequences of the 6 biotinylated EWS::FLI1 capture probes; shRNA clone IDs (Sigma SHC002, TRCN0000005322); Addgene plasmid RRIDs. Methods - 'Lentivirus Production and Genetic knockdown', 'EWS::FLI1 long read sequencing'.	

Cell materials	Indicate where provided: section/figure legend	N/A
Cell lines: Provide species information, strain. Provide accession number in repository OR supplier name, catalog number, clone number, OR RRID.	Key Resources Table: species, source (ATCC / COG / ref. 21) and Cellosaurus RRID for every line, incl. 293FT. Methods - 'Cell Lines and PDX': culture conditions, STR authentication every 6 months, mycoplasma testing. Fig. 1-figure supplement 1A: lineage, patient age/sex, fusion type and tumour site for all 14 lines.	
Primary cultures: Provide species, strain, sex of origin, genetic modification status.	Human bone-marrow mesenchymal stem cells (5046L, 240L, H7; not genetically modified): Key Resources Table and Methods - 'Cell Lines and PDX' (source); Fig. 1-figure supplement 1A (donor age/sex).	

Experimental animals	Indicate where provided: section/figure legend	N/A
Laboratory animals or Model organisms: Provide species, strain, sex, age, genetic modification status. Provide accession number in repository OR supplier name, catalog number, clone number, OR RRID.	No new animal experiments were performed. Xenograft data in Fig. 5H are re-analysed from a published study (ref. 21): CHLA10 xenografts in NSG mice, subcutaneous or tail-vein injection - Methods - 'Digital Spatial Profiling'; Fig. 5H legend (strain, injection route); further animal details in ref. 21.	
Animal observed in or captured from the field: Provide species, sex, and age where possible.		N/A

Plants and microbes	Indicate where provided: section/figure legend	N/A
Plants: provide species and strain, ecotype and cultivar where relevant, unique accession number if available, and source (including location for collected wild specimens).		N/A
Microbes: provide species and strain, unique accession number if available, and source.		N/A

Human research participants	Indicate where provided: section/figure legend) or state if these demographics were not collected	N/A
If collected and within the bounds of privacy constraints report on age, sex, gender and ethnicity for all study participants.	Fig. 5-fig. suppl. 1D (age, sex, site; 6 snRNA-seq tumours); Fig. 1-fig. suppl. 1A (cell-line donor age/sex). Public cohorts: gender and ethnicity not collected.	

Design:

Study protocol	Indicate where provided: section/figure legend	N/A
If the study protocol has been pre-registered, provide DOI. For clinical trials, provide the trial registration number OR cite DOI.	Not pre-registered; not a clinical trial.	N/A

Laboratory protocol	Indicate where provided: section/figure legend	N/A
Provide DOI OR other citation details if detailed step-by-step protocols are available.	Methods - 'Cell and library prep' (10x CG000338 Rev C); 'EWS::FLI1 long read sequencing' (10x CG000315 Rev B, IDT xGen v2, PacBio SMRTbell 3.0; ref. 68).	

Experimental study design (statistics details) *		
**For in vivo studies: State whether and how the following have been done**	**Indicate where provided: section/figure legend. If it could have been done, but was not, write "not done"**	**N/A**
Sample size determination	Not done - no formal sample-size calculation; no new in vivo experiments.	
Randomisation	Not done - no new in vivo experiments; cell-line experiments were not randomised.	
Blinding	Not done - no new in vivo experiments; computational analyses were not blinded.	
Inclusion/exclusion criteria	Cell-level QC: Methods - 'Single-cell multiome data analysis', 'scRNAseq Patient Tumors'; Fig. 1-fig. suppl. 1B, Fig. 3-fig. suppl. 1A, Fig. 5-fig. suppl. 1A.	

Sample definition and in-laboratory replication	Indicate where provided: section/figure legend	N/A
State number of times the experiment was replicated in the laboratory.	Methods - 'Cell and library prep' (cell lines pooled 1:1:1 per capture). Single-cell datasets: 14 lines (Fig. 1A); shFLI1 vs shNS, 4 lines (Fig. 3D); TGF-beta vs vehicle, 3 lines (Fig. 5A-C); cohort n in Fig. 6 legend.	
Define whether data describe technical or biological replicates.	Biological replicates = distinct cell lines / PDXs / patient tumours (Fig. 1A, 3D, 5A-C, 6A-B legends); unit of analysis = single cells.	

Ethics	Indicate where provided: section/submission form	N/A
Studies involving human participants: State details of authority granting ethics approval (IRB or equivalent committee(s), provide reference number for approval.	Methods - 'Study approval': Fred Hutchinson Cancer Center IRB, protocol FHIRB0010627; no participants recruited, patient data de-identified and previously published (consent statement included); also in submission form.	
Studies involving experimental animals: State details of authority granting ethics approval (IRB or equivalent committee(s), provide reference number for approval.	No new animal experiments were performed in this study; xenograft DSP data were re-analysed from ref. 21 (Methods - 'Digital Spatial Profiling').	N/A
Studies involving specimen and field samples: State if relevant permits obtained, provide details of authority approving study; if none were required, explain why.	No field samples. Human tumour specimens/data are covered by the IRB approval above or come from published, de-identified public datasets (refs. 9, 49-51).	N/A

Dual Use Research of Concern (DURC)	Indicate where provided: section/submission form	N/A
If study is subject to dual use research of concern regulations, state the authority granting approval and reference number for the regulatory approval.		N/A

Analysis:

Attrition	Indicate where provided: section/figure legend	N/A
Describe whether exclusion criteria were pre-established. Report if sample or data points were omitted from analysis. If yes, report if this was due to attrition or intentional exclusion and provide justification.	Pre-specified cell-level QC filters (mitochondrial reads <15%, RNA UMI range, ATAC quality, doublets): Methods - 'Single-cell multiome data analysis', 'scRNAseq Patient Tumors'; Fig. 1-fig. suppl. 1B, Fig. 3-fig. suppl. 1A, Fig. 5-fig. suppl. 1A. Survival analysis used the 46 uniformly treated GSE63157 tumours with outcome data (Results).	

Statistics	Indicate where provided: section/figure legend	N/A
Describe statistical tests used and justify choice of tests.	Methods - 'Statistics' (Wilcoxon rank-sum, BH correction, Pearson, Cox model); thresholds and n in legends (Fig. 1F-G, 2C, 2E, 3F-H, 4C-E, 5I, 6E-H).	

Data availability	Indicate where provided: section/submission form	N/A
For newly created and reused datasets, the manuscript includes a data availability statement that provides details for access (or notes restrictions on access).	Methods - 'Data and materials availability' (new data: GEO GSE332630, Zenodo 10.5281/zenodo.12209095, github.com/furlan-lab/EwS_multiome); reused data: Methods - 'External Data', 'scRNAseq Patient Tumors', 'Digital Spatial Profiling'; also in submission form.	
When newly created datasets are publicly available, provide accession number in repository OR DOI and licensing details where available.	GEO: GSE332630 (public; raw sequence data and processed data). Processed Seurat objects: Zenodo, doi:10.5281/zenodo.12209095 (MIT licence). Analysis code: https://github.com/furlan-lab/EwS_multiome. No restrictions on access.	
If reused data is publicly available provide accession number in repository OR DOI, OR URL, OR citation.	Methods - 'External Data', 'scRNAseq Patient Tumors', 'Digital Spatial Profiling': GEO GSE236289, GSE63157; PRJNA253759, PRJNA261990; ScPCA (ref. 49); PDX scRNA-seq (19); DSP data (21); EWS::FLI1 binding sites (38).	

Code availability	Indicate where provided: section/figure legend	N/A
For any computer code/software/mathematical algorithms essential for replicating the main findings of the study, whether newly generated or re-used, the manuscript includes a data availability statement that provides details for access or notes restrictions.	Methods - 'Data and materials availability': all analysis code is available at https://github.com/furlan-lab/EwS_multiome without restriction. Reused software and packages are listed with RRIDs / URLs in the Key Resources Table and cited in Methods - 'Single-cell analysis', 'External Data' and 'Deconvolution'.	
Where newly generated code is publicly available, provide accession number in repository, OR DOI OR URL and licensing details where available. State any restrictions on code availability or accessibility.	https://github.com/furlan-lab/EwS_multiome (analysis notebooks for all figures); https://github.com/furlan-lab/piperna (bulk RNA-seq alignment workflow); both listed in the Key Resources Table. Processed objects: Zenodo doi:10.5281/zenodo.12209095 (MIT licence). No restrictions on access.	
If reused code is publicly available provide accession number in repository OR DOI OR URL, OR citation.	Key Resources Table (RRIDs / URLs): Cell Ranger ARC, ArchR (37), Signac (65), Seurat (46), Souporcell (64), CellBender, chromVAR, HOMER (43), clusterProfiler, decoupleR (47), STAR (69), oligo, IsoSeq3, pbfusion, cellSignalAnalysis (52).	

Reporting:

The MDAR framework recommends adoption of discipline-specific guidelines, established and endorsed through community initiatives.

Adherence to community standards	Indicate where provided: section/figure legend	N/A
State if relevant guidelines (e.g., ICMJE, MIBBI, ARRIVE, STRANGE) have been followed, and whether a checklist (e.g., CONSORT, PRISMA, ARRIVE) is provided with the manuscript.	MDAR checklist provided. Author contributions reported using CRediT-style roles ('Author contributions'). ARRIVE, CONSORT and PRISMA checklists are not applicable (no new animal experiments, clinical trial or systematic review).	

---

* We provide the following guidance regarding transparent reporting and statistics; we also refer authors to Ten common statistical mistakes to watch out for when writing or reviewing a manuscript.

Sample-size estimation

- You should state whether an appropriate sample size was computed when the study was being designed
- You should state the statistical method of sample size computation and any required assumptions
- If no explicit power analysis was used, you should describe how you decided what sample (replicate) size (number) to use

Replicates

- You should report how often each experiment was performed
- You should include a definition of biological versus technical replication
- The data obtained should be provided and sufficient information should be provided to indicate the number of independent biological and/or technical replicates
- If you encountered any outliers, you should describe how these were handled
- Criteria for exclusion/inclusion of data should be clearly stated
- High-throughput sequence data should be uploaded before submission, with a private link for reviewers provided (these are available from both GEO and ArrayExpress)

Statistical reporting

- Statistical analysis methods should be described and justified
- Raw data should be presented in figures whenever informative to do so (typically when N per group is less than 10)
- For each experiment, you should identify the statistical tests used, exact values of N, definitions of center, methods of multiple test correction, and dispersion and precision measures (e.g., mean, median, SD, SEM, confidence intervals; and, for the major substantive results, a measure of effect size (e.g., Pearson's r, Cohen's d)
- Report exact p-values wherever possible alongside the summary statistics and 95% confidence intervals. These should be reported for all key questions and not only when the p-value is less than 0.05.

Group allocation

- Indicate how samples were allocated into experimental groups (in the case of clinical studies, please specify allocation to treatment method); if randomization was used, please also state if restricted randomization was applied
- Indicate if masking was used during group allocation, data collection and/or data analysis
