# Supplementary material for: Multimodal single-cell analyses reveal distinct fusion-regulated transcriptional programs in Ewing sarcoma
Source: eLife. 2026 Nov 11;14:RP109124. doi: 10.7554/eLife.109124 (PMC13754278; doi:10.7554/eLife.109124)
Supplement: Supplementary file 2 [file elife-109124-fig1-data1.zip › Figure 1-source data 1/Figure 1E_uncropped_labelled.pdf]

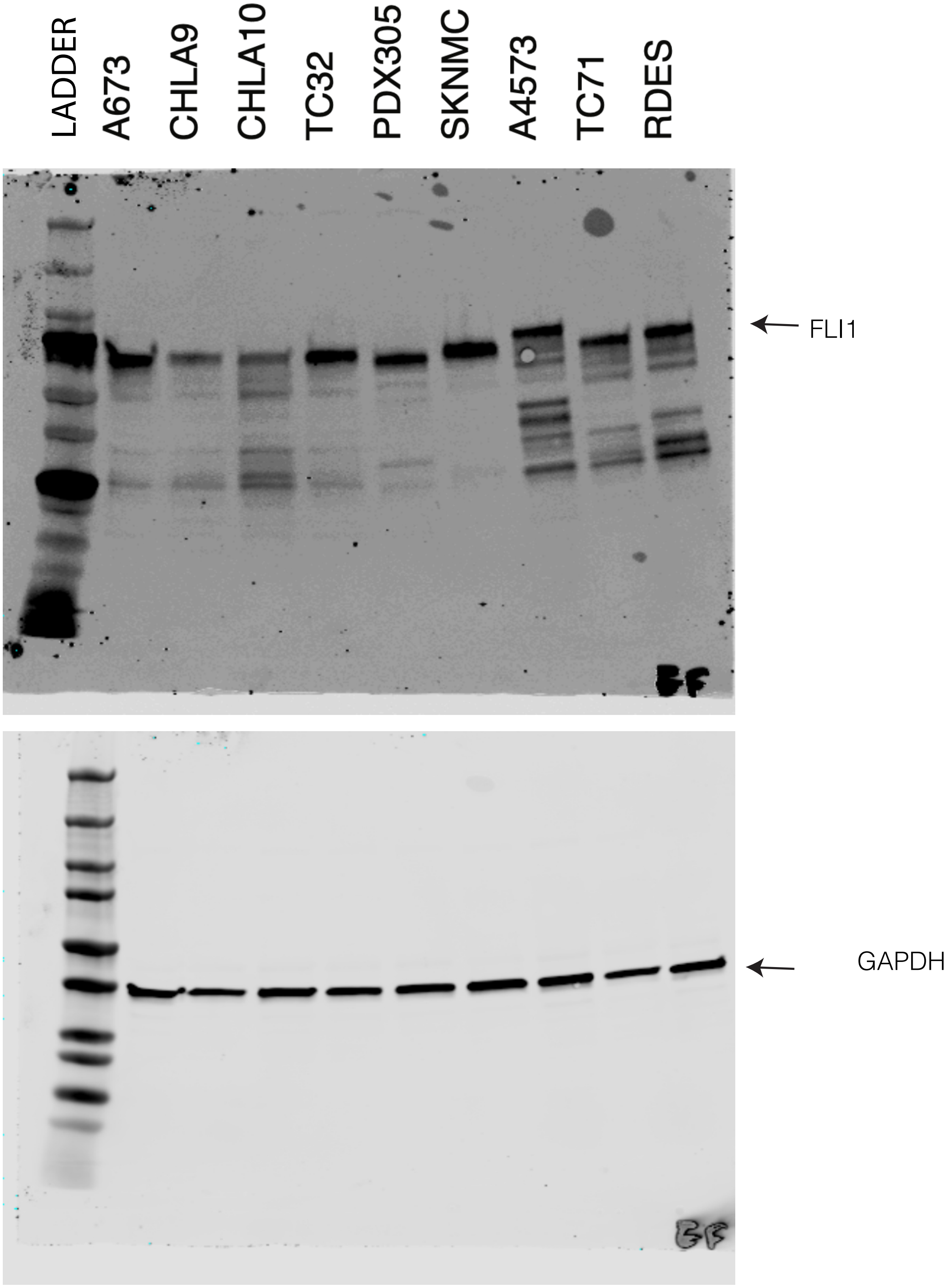
LADDER
A673
CHLA9
CHLA10
TC32
PDX305
SKNMC
A4573
TC71
RDES
FLI1
GAPDH
