# Supplementary material for: Multimodal single-cell analyses reveal distinct fusion-regulated transcriptional programs in Ewing sarcoma
Source: eLife. 2026 Nov 11;14:RP109124. doi: 10.7554/eLife.109124 (PMC13754278; doi:10.7554/eLife.109124)
Supplement: Supplementary file 4 [file elife-109124-fig2-data1.zip › Figure 2-source data 1/Figure 2G_uncropped_labelled.pdf]

GEL1 scans (chronological)

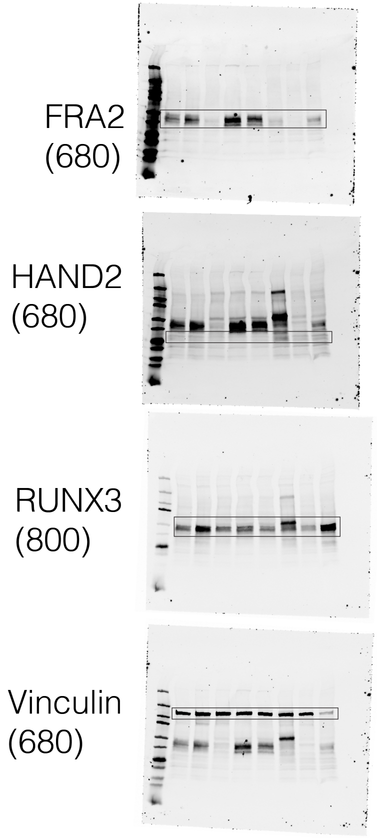

GEL2 scans (chronological)

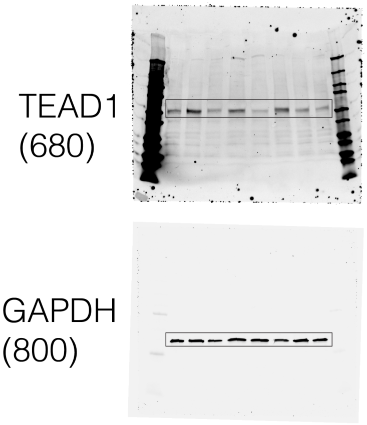

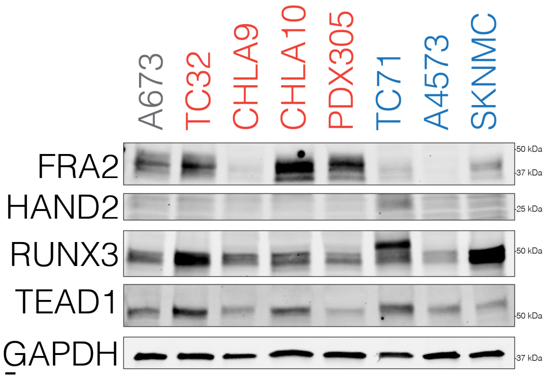

Primaries & predicted weights

RUNX3 ms 13089T ~45 kDa gel1
Fra2 Rbt 19967T~35-45 kDa gel1
HAND2 Gt AF3876 ~25 kDa gel1
Rbt 13901S Vinculin ~120 kDa (both)
Rbt 12292T TEAD1 ~50 kDa gel2
GAPDH Ms AM4300 ~37 kDa
Rbt 5281TFra1 ~35-40 kDa gel2
