# Supplementary material for: Multimodal single-cell analyses reveal distinct fusion-regulated transcriptional programs in Ewing sarcoma
Source: eLife. 2026 Nov 11;14:RP109124. doi: 10.7554/eLife.109124 (PMC13754278; doi:10.7554/eLife.109124)

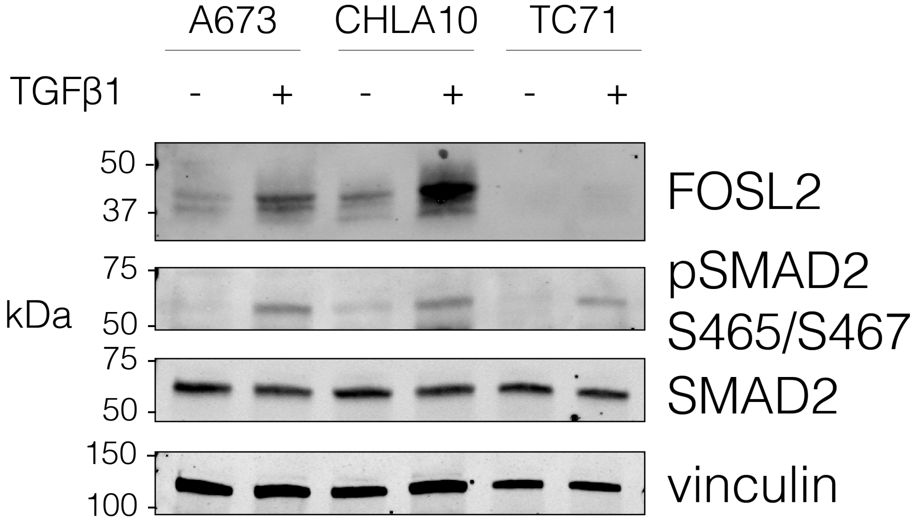


precision plus dual color protein ladder

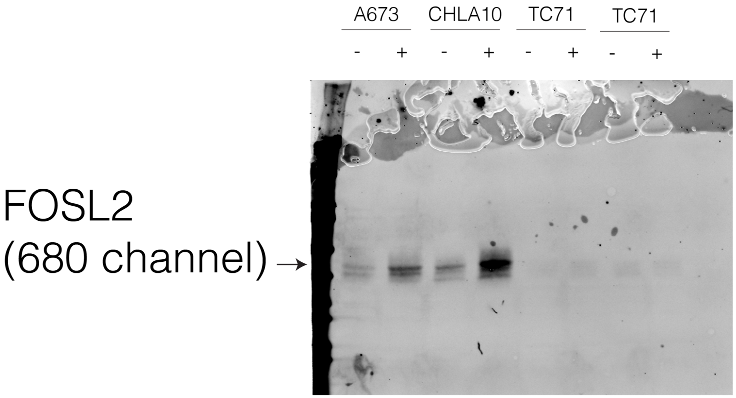


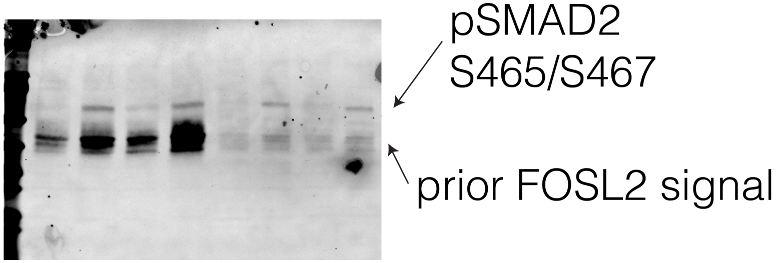


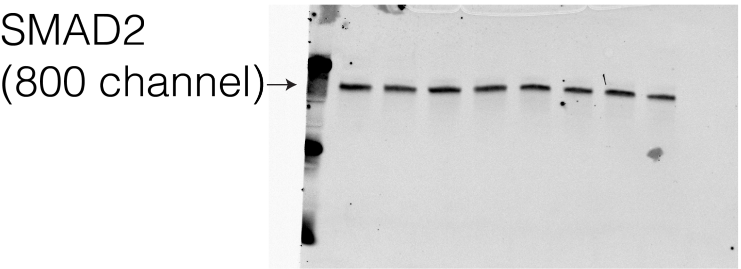


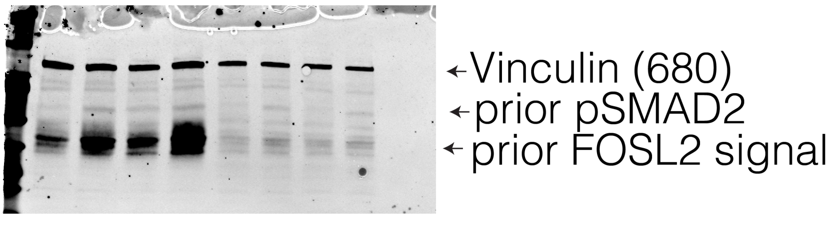

Supplement: Supplementary file 6 [file elife-109124-fig5-data1.zip › Figure 5-source data 1/Figure 5G_uncropped_labelled.pdf]
