# Supplementary material for: Multimodal single-cell analyses reveal distinct fusion-regulated transcriptional programs in Ewing sarcoma
Source: eLife. 2026 Nov 11;14:RP109124. doi: 10.7554/eLife.109124 (PMC13754278; doi:10.7554/eLife.109124)
Supplement: Peer review history

# Editor report: eLife Assessment

**DOI:** 10.7554/eLife.109124.3.sa0

**Authors:** Lynne-Marie Postovit

**Affiliations:** Queens University Canada

**Keywords:** Compelling, Valuable

This **valuable** study presents an analysis of the gene regulatory networks that contribute to tumour heterogeneity and tumor plasticity in Ewing sarcoma, with key implications for other fusion-driven sarcomas. The authors employed **compelling** orthogonal approaches, including single-cell sequencing and xenografts, to reveal the existence and plasticity of specific gene regulatory networks (e.g., TGF-beta signaling) within Ewing sarcoma, as well as significant differences that exist between cell lines and patient tumors.

# Referee report: Reviewer #1 (Public review)

**DOI:** 10.7554/eLife.109124.3.sa1

The investigators elegantly utilized single-cell co-assay of RNA and ATAC seq to unveil the heterogeneous gene regulatory networks in Ewing sarcoma. The authors should be commended on their ability to identify multiple unique modules of gene regulation of Ewing sarcoma utilizing complex computational methods between numerous Ewing sarcoma cell lines. Additionally, they complimented their single cell findings with xenografts as well as primary Ewing sarcoma patient tumors - validating the intratumoral heterogeneous gene regulatory networks of Ewing sarcoma. More importantly, they have revealed that exogenous TGF-B may modify these distinct epigenetic and transcriptional signatures within Ewing sarcoma tumors. Overall, the manuscript highlights an important discovery of the heterogenous gene regulatory programming of Ewing sarcoma and further highlights the role that TGFB plays within the tumor microenvironment of Ewing sarcoma. There are some areas of ambiguity that require clarification to increase the impact of the manuscript.

Comments on the latest revision:

The responses to my review were appropriate and my comments were all addressed.

# Referee report: Reviewer #2 (Public review)

**DOI:** 10.7554/eLife.109124.3.sa2

Summary:

This work by Waltner, et. al. provides a comprehensive single cell multiomics analysis of plasticity in gene regulatory networks present in Ewing sarcoma using single cell RNA-sequencing (scRNA-seq) and single cell assay for transposase accessible chromatin with sequencing (scATAC-seq). They find that Ewing sarcoma cell lines models have distinct patterns of chromatin accessibility compared to non-Ewing sarcoma models, and that there is significant variability across Ewing sarcoma cell lines, and sometimes within a single cell line. These differences across models are linked to 3 distinct gene regulatory modules, 2 of which are present across the range of model systems studied here. The first modules present across models is activated when the fusion is expressed and includes genes enriched for the known EWSR1::FLI1 response element, GGAA microsatellites along with other neural crest transcription factors. The other module primarily consists of genes repressed by EWSR1::FLI1, which are activated in EWSR1::FLI1-low states. Interestingly, EWSR1::FLI1-low cells have already been tied to more migratory and metastatic phenotypes and the data here suggest these cells are more responsive to external signals from TGF-β and this may be mediated through FOSL2-mediated gene regulation. This is a technically rigorous study, with a variety of different analytical techniques used to address similar questions and this approach elevates confidence in the answers provided. This is further strengthened by the diverse set of model systems used, including patient-derived cell lines, cell line xenograft models, patient-derived xenografts, mining available single cell data from patient samples, and validation of the gene modules identified in a larger set of patient microarray samples. In whole, this study provides a valuable resource for understanding heterogeneity, plasticity, and gene expression networks in Ewing sarcoma. This may be a useful resource for future studies of metastatic disease and provide a framework for similar questions in other fusion-driven sarcomas.

Comments on revised version.

The authors have addressed comments from my prior review. Thank you!

# Author comment: Author response

**DOI:** 10.7554/eLife.109124.3.sa3

**Authors:** Olivia G Waltner, April A Apfelbaum, Emma D Wrenn, Shruti S Bhise, Sami B Kanaan, Rula Green Gladden, Mark A Mendoza, Roger Volden, Zev Kronenberg, Anand G Patel, Michael A Dyer, Jay F Sarthy, Elizabeth R Lawlor, Scott Furlan

**Affiliations:** Fred Hutch Cancer Center Seattle United States; Seattle Children's Research Institute Seattle United States; Seattle Children's Research Institute Seattle United States; Fred Hutch Cancer Center Seattle United States; Fred Hutch Cancer Center Seattle United States; Fred Hutch Cancer Center Seattle United States; Fred Hutch Cancer Center Seattle United States; Pacific Biosciences Menlo Park United States; Pacific Biosciences Menlo Park United States; St. Jude Children's Research Hospital Memphis United States; St Jude Children's Research Hospital Memphis United States; Seattle Children's Research Institute Seattle United States; Seattle Children's Research Institute Seattle United States; Fred Hutch Cancer Center Seattle United States

The following is the authors’ response to the original reviews.

**Public Reviews:**

We thank both reviewers for their thoughtful and constructive evaluations of our manuscript. We are pleased that the reviewers recognize the value of our multimodal single-cell approach and the diversity of model systems used to define gene regulatory networks underlying heterogeneity in Ewing sarcoma.

**Reviewer #1 (Public review):**

The investigators elegantly utilized a single-cell co-assay of RNA and ATAC seq to unveil the heterogeneous gene regulatory networks in Ewing sarcoma. The authors should be commended on their ability to identify multiple unique modules of gene regulation of Ewing sarcoma utilizing complex computational methods between numerous Ewing sarcoma cell lines. Additionally, they complemented their single-cell findings with xenografts as well as primary Ewing sarcoma patient tumors - validating the intratumoral heterogeneous gene regulatory networks of Ewing sarcoma. More importantly, they have revealed that exogenous TGF-β may modify these distinct epigenetic and transcriptional signatures within Ewing sarcoma tumors. Overall, the manuscript highlights an important discovery of the heterogenous gene regulatory programming of Ewing sarcoma and further highlights the role that TGFB plays within the tumor microenvironment of Ewing sarcoma. There are some areas of ambiguity that require clarification to increase the impact of the manuscript.

We appreciate Reviewer 1's positive assessment of our work and their recognition of the importance of identifying heterogeneous gene regulatory programs in Ewing sarcoma, including the role of TGF-β in the tumour microenvironment. We have addressed the areas of ambiguity noted by the reviewer, including clarifying cluster assignments and the selection of k=3 for module identification, adding statistical comparisons to relevant figures, correcting figure cross-references, and improving figure labeling for clarity. We have also added higher-resolution images of the spatial profiling data and highlighted relevant correlations between CHLA9 and CHLA10 clusters. We believe these revisions improve the clarity and rigour of the manuscript.

**Reviewer #2 (Public review):**

Summary:

This work by Waltner et. al. provides a comprehensive single-cell multiomics analysis of plasticity in gene regulatory networks present in Ewing sarcoma using single-cell RNA-sequencing (scRNA-seq) and single-cell assay for transposase accessible chromatin with sequencing (scATAC-seq). They find that Ewing sarcoma cell line models have distinct patterns of chromatin accessibility compared to non-Ewing sarcoma models, and that there is significant variability across Ewing sarcoma cell lines, and sometimes within a single cell line. These differences across models are linked to 3 distinct gene regulatory modules, 2 of which are present across the range of model systems studied here. The first modules present across models are activated when the fusion is expressed and include genes enriched for the known EWSR1::FLI1 response element, GGAA microsatellites, along with other neural crest transcription factors. The other module primarily consists of genes repressed by EWSR1::FLI1, which are activated in EWSR1::FLI1-low states. Interestingly, EWSR1::FLI1-low cells have already been tied to more migratory and metastatic phenotypes, and the data here suggest these cells are more responsive to external signals from TGF-β, and this may be mediated through FOSL2-mediated gene regulation. While there are some minor additional validation studies that can be performed to strengthen a few individual analyses, this is a technically rigorous study, with a variety of different analytical techniques used to address similar questions, and this approach elevates confidence in the answers provided. This is further strengthened by the diverse set of model systems used, including patient-derived cell lines, cell line xenograft models, patient-derived xenografts, mining available single-cell data from patient samples, and validation of the gene modules identified in a larger set of patient microarray samples. In whole, this study provides a valuable resource for understanding heterogeneity, plasticity, and gene expression networks in Ewing sarcoma. This may be useful for future studies of metastatic disease and may also provide a framework for similar questions in other fusion-driven sarcomas.

Strengths:

There are a few core strengths in this study. First is the number and diversity of Ewing sarcoma models studied, spanning commonly used cell lines, patient-derived xenografts, and patient samples. The second is the large array of rigorous and orthogonal approaches used to uncover the identity and function of various gene modules. This includes an array of informatics techniques, as well as specific modulation of cell line models in culture. A third is confirmation that different gene expression programs are present in the same tumor using spatial transcriptomic analysis. Lastly, the authors have made all of their data and code accessible, enabling continued use of this dataset as a resource for others.

Weaknesses:

As highlighted by the authors, this study is somewhat limited by the small number of single-cell data from patient samples that are publicly available. Much of the analysis comes from cell lines. Additionally, they focus only on one type of signal that may modulate cell plasticity, and there are likely to be many others. Lastly, there are a few weak spots in the data. Some of this likely arises from the underlying complexity of the data, the generally sparse nature of scATAC data, and the biological heterogeneity present in the cell lines studied. The most pronounced weakness was in the analysis of transcription factors that dictate gene expression in the distinct modules, as well as the response to TGF-β. While some specific transcription factors showed module-specific expression consistent with the computational prediction in Figure 2, others did not likely due to additional factors not tested here. Likewise, the same transcription factors did not always show consistent enrichment in the gene modules that responded to TGF-β treatment when analyzed across cell lines. On the whole, these are relatively minor weaknesses and do not diminish the value of this study.

We thank Reviewer 2 for their thorough and balanced assessment. We agree that the study's strengths lie in the breadth of model systems and orthogonal analytical approaches, and we appreciate the reviewer's acknowledgement that the identified weaknesses are relatively minor.

In response to the reviewer's suggestions, we have made several substantive improvements. First, we have included a new Western blot panel in Figure 1 showing EWS::FLI1 protein levels across cell lines, which provides important context for interpreting the long-read fusion transcript detection data. Second, we have revised text throughout the Results section to improve precision — in particular, clarifying that our chromatin analyses assess accessibility at published EWS::FLI1 binding sites rather than binding per se, and ensuring that our stated hypotheses match the metrics presented in the corresponding figures. Third, we have improved figure color schemes and labeling to aid interpretation and corrected errors in panel labeling.

Regarding the reviewer's observation about transcription factor enrichment patterns across cell lines (particularly RUNX3 in the TGF-β response analysis and FOSL2’s modest expression at the protein level in CHLA9), we acknowledge that not all TFs showed perfectly consistent module-specific behaviour across every cell line. As the reviewer notes, this likely reflects the underlying biological complexity and additional regulatory factors not tested here. We have made attempts to temper our language accordingly.

We believe that the revised manuscript, with its additional experimental data, improved figures, and clarified text, addresses the concerns raised by both reviewers and strengthens the overall impact of our findings.

**Recommendations for the authors:**

**Reviewer #1 (Recommendations for the authors):**

Specific comments:

(1) Figure 1A: Suggest adding cell labels on the UMAP plot - difficult to tell with different colors - which cluster is which cell line.

We have added labels to Figure 1A (UMAP plot) as suggested.

(2) Figure 1B: Although this may have been slightly addressed later in the manuscript, since CHLA9 and 10 are from the same patient, are the authors surprised to see the differences in EWS::FLI1 motif accessibility, or are these findings further reinforcement of inherent heterogeneity? Should one expect the ChromVAR deviation z-score at least to overlap between CHLA9 and 10, since they're from the same patient, if not, can the authors explain why?

We were indeed surprised to see differences in EWS::FLI motif accessibility inferred from our data particularly from the isogenic lines CHLA9 and 10. While it is impossible to know for sure, we suspect that some treatment related changes increased accessibility in CHLA10. However, when considering global accessibility signatures, a subset of cells from CHLA10 (C23) was most correlated with signatures from CHLA9. We have addressed this by stating “Unsurprisingly, of all cell lines, CHLA10 C23 cells exhibited the greatest correlation to profiles from CHLA9,” in the 11th paragraph of the results section.

(3) Figure 1B: Can the authors comment on the differences in trends of fusion transcript to chromatin enrichment (e.g., A673 and TC71 inverse trends vs. other cell lines that are direct positive or negative trends).

We are hesitant to draw too many conclusions from transcript counting vs chromatin enrichment given some outliers, but in general, we found that high-fusion transcribing cell lines A4573, SKNMC, RDES were associated with module 3, while the lower transcribing cells CHLA9/10, TC32 and PDX305 were associated with module 2. We did not choose to emphasize this correlation too strongly in the paper as there may be other factors such as additional mutations (such as BRAF^V600E^ in A673) that may have either been present in the original tumour or after serial passaging that may play a role.

(4) Figure S1D: Please make the pt. smaller in order to better visualize the differences and highlight the stark differences of the ChromVAR binding site score.

We have adjusted the point size of the embeddings in Fig S1D to enhance visualization.

(5) Figure S1D: Is it surprising to see a large proportion of PDX305 and minor proportions of CHLA10 and CHLA9 with low ChromVAR binding site score?

Our analyses indeed show that PDX305, CHLA9, and CHLA10 utilise fusion-repressed gene programs, so lower enrichment of EWS: FLI1 microsatellites is consistent with our findings.

(6) Figure 2A: Unclear how k = 3 was ultimately selected - was it purely a visualization of how well separated each of the cell lines is? Can the authors further clarify how k = 3 was determined to be the most optimal? Is there a UMAP that demonstrated a difference in clustering between groups 1, 2, and 3? Is there a threshold cut-off to assign groups into 1, 2, or 3?

Thank you for pointing out this omission. In Fig 1 we show that unsupervised clustering of DA peaks across cell lines grouped EwS cell lines into 2 clusters. When using peak-2-gene linkages (Fig 2), we chose a k = 3 to explore the potential genes/CREs explaining the grouping found in Fig 1 because A673 was a clear outlier. We have added the following sentence to the results section paragraph 6. “We selected k = 3 to extend the two-cluster structure observed in Fig. 1F–G, reasoning that A673 represented a clear outlier whose distinct regulatory program would be obscured at lower k.”

(7) Figure 2G: Understanding the substantial heterogeneity of each cell and TF binding motifs - given that CHLA9 is considered group 2, is it unexpected that FOSL2 was not highly expressed compared to the other EwS cell lines within group 2: (TC32, CHAL10, PDX305).

Thank you for astutely pointing out that CHLA9 did defy the trend for FOSL2 protein expression compared with other group 2 lines. We specifically did not comment on this finding in the manuscript as we could not account for its status as an outlier. We address this in the public response above.

(8) Figure S3B: Did the authors perform a similar computational analysis (performed for Figure 4), looking specifically at CHLA9 (Clusters 24 and 25) to determine if there are any overlaps with CHLA10 cluster 23's pathway activity/MSigDB/GO: Biological process terms? If there are potential overlaps, can the authors potentially infer tumor clonal evolution from CHLA9 to CHLA10?

While we didn’t do pathway analysis, Figure S3 shows strong pearson correlation of C23 from CHLA10 with both C24 and C25 from CHLA9. We have highlighted this with a red box in the figure to make this more obvious to the reader.

(9) Figure 4: Given the heterogeneity of CHLA-10. Did the authors observe any differences in morphology within CHLA-10 between the predominant modules (modules 2 and 3), given such stark transcriptional heterogeneity?

We did not observe any morphologic differences within CHLA10 but acknowledge this would be an interesting avenue of further investigation.

(10) Figure 5B: Can the authors also plot out module 3 gene expression to see if the cluster enrichment is unique from module 2 gene expression with TGFB1 and vehicle?

We thank the reviewer for this suggestion and have replaced Fig S4B with violin plots so the enrichments are clearer. Module 3 expression in cluster 3 cells from CHLA10 is among the lowest in the cell line.

(11) Figure 5H: Can the authors provide a higher zoom/resolution of the H&E stain of the ROIs in order to see if there are indeed more stroma/fibrosis in ROI9 and ROI10, and if there are differences in tumor cell morphology within different ROIs that harbor different modules?

We have now included higher resolution and magnification of IHC panels in Figure 5H. These images are included as Supplemental Fig 4C. It is notable that although no dramatic differences in tumour cell morphology are visualized, ROI9 and ROI10 comprise small islands of viable tumour surrounded by necrosis.

(12) Figure 6: Within Volchenboum/Lawlor's dataset, of the 46 clinically annotated primary tumors, 10 of the COG samples contained substantial stromal elements, while all the tumors in the European cohort were >70% viable tumors. Can the authors separate out the stromal-rich (n = 10) samples and analyze the 36 tumor-enriched samples to see if the survival curve is the same as what is shown in Figure 6G/H and S4E?

We thank the reviewer for this suggestion. We observed no differences when stratifying the patients as outlined. Indeed, in the original Volchenboum et al, manuscript it was demonstrated that no prognostic gene signature was identifiable when the stromal-rich tumours were removed from the cohort.

(13) Figure 6: Additionally, can the authors run a similar computational analysis to determine the predominant modules within bulk sequencing of the Volchenboum/Lawlor dataset between each tumor?

The computational approach deconvolution was developed for use with bulk RNA-seq and is based on count data. The linear mixture model at the heart of deconvolution methods requires that the measured signal is proportional to abundance across the full range, and Affymetrix microarray data violate that assumption in a gene-specific, nonlinear way that can't be fully corrected post hoc.

(14) Page 15, line 2: Wrong GSE data cited - currently cited as GSE61357 - should be GSE63157 instead.

This has been corrected, thank you.

(15) Please add statistical comparisons for Figure 3E-H.

We thank the reviewer for highlighting this omission. We used the software package ggpubr to perform Wilcoxon rank-sum tests comparing mean module scores between conditions. We have added statistical labels to the plots. All comparisons were statistically to the level indicated in the figure.

(16) Page 11 Line 21: Figure S3 D-E is not about EMT, migration, and TGFB signaling - I believe the authors are referring to Figures 4D-E

Thank you, we have corrected these errors

**Reviewer #2 (Recommendations for the authors):**

(1) Data

(a) In Figure 1 and the associated text, there is an analysis of cells expressing EWSR1::FLI1 performed using a locus-specific amplification and long-range sequencing. On page 7, lines 1-5, there is some discussion about how some of these track with overall transcript levels, while others don't. Additionally, a very low fraction of cells is shown to be EWSR1::FLI1 positive. This analysis might also be strengthened by a Western blot to show protein levels and how they vary across the cell lines, which may help explain additional differences in the data. The Abcam antibody ab133485 works well for western blotting of EWSR1::FLI1. While this isn't additional single-cell data, the percentage of cells with transcript detected is not really equivalent to the total expression level. This seems particularly valuable to do, as prior publications (Pishas, et. al., Mol. Cancer. Ther., 2018) show that of the cell lines tested here, TC32 has relatively high EWSR1::FLI1 protein levels, while A673 has relatively low expression. This contrasts with the percent of positive cells here.

We thank the reviewer for this suggestion and have included a new panel, Figure 1E containing the western results for cells harvested in log-phase growth.

(b) Figures 5D-F are not obviously referenced in the section about Figure 5 (page 12 line 4 through pg. 13 line 20). One question to pose to the authors is how to interpret the data here for TC71 in light of the fact that they were relatively insensitive to TGF-β. This shows up obviously in the Western blot in 5G.

Thank you, for drawing attention to this omission. We have corrected the figure reference to include Figure 5D-F. In regards to our interpretation of TC71’s lack of response to TGF-B, we direct the authors attention to our statement: “The muted response of the TC71 cell line (module-3 dominant) to the influence of TGF-b (Fig. 5A-C, Fig. S4B) suggests that pre-existing transcriptional states may condition the sensitivity to TGF-β signalling.”

(c) For the discussion of Figure 5, the authors say on page 12, line 21, that "RUNX3 was enriched in non-responsive clusters." I'm not entirely convinced that the data support this statement as written. This is true in A673s, but there appears to be no difference between the maximally responsive and maximally non-responsive clusters in CHLA10. TC71 was not particularly responsive, but showed the opposite effect.

We agree this was not clearly written. We have de-emphasized RUNX3 findings here. Our new conclusion in the results section paragraph 15 is: “Correlation analyses revealed a reciprocal enrichment pattern of many key TFs from Figure 2, where correlation of FOSL2 (but not RUNX3) gene expression and accessibility was generally highest in TGF-β responsive clusters.”

Can data for A673 cells be included for Figure 5G? Like CHLA10, this cell line had the pattern of FOSL2 enrichment that is concordant with that described in the text.

We thank the reviewer for this suggestion and have included A673 in the western blot.

(2) Figures

(a) In Figure 2A, it is very difficult to distinguish the colors for CHLA9 and TC32 in the left panel. Can the color scheme here be changed to make these easier to distinguish?

We thank the reviewer for pointing this out and we have added labels to the UMAP. We hope this makes the visualization more obvious.

(b) Similarly, the KLF4 and SP1 lines in Figure 2D are a little close and might benefit from having more distinct colors.

We thank the reviewer for pointing this out. We have changed the KLF4 to a green hue.

(c) The lower panel of Figure 5I has 2 samples labeled "11" and no sample labeled "12".

Thank you for catching this error. It has been corrected.

(3) Text

(a) One section early in the response was a little bit confusing and could benefit from some revision to improve clarity. On page 6, lines 9-11, this reads a little bit like they looked at cell-line specific EWSR1::FLI1 binding, but that wasn't the assay that was performed. Perhaps there is a better way to describe this than simply "enrichment of EWS::FLI1 sites."

We thank the reviewer for their efforts to improve clarity of our work.

We changed the following sentence in the 2nd paragraph of the result section:

"We visualized the enrichment of EWS::FLI1 sites across all cell lines and discovered distinct EwS cell-line specific usage (Fig. 1C & Fig. S1D)."

And revised to:

"We then assessed chromatin accessibility at these published EWS::FLI1 binding sites across all cell lines and discovered that accessibility at these loci varied in a cell-line-specific manner (Fig. 1C & Fig. S1D)."

(b) Then at the start of the next paragraph (page 6 line 12), the authors talk about "differences in EWS::FLI1 motif and binding site enrichment" and my first thoughts were whether this was differences in which sites were bound or differences in the strength of enrichment. More precise language would be helpful.

Here in the 3rd paragraph of the result section we changed:

"Given the differences in EWS::FLI1 motif and binding site enrichment"

To:

"Given the heterogeneity in the magnitude of EWS::FLI1 motif enrichment"

(c) Related to the comment about EWSR1::FLI1 positive cells vs. protein levels above, the hypothesis on page 6, line 13 says that there was a hypothesis that different Ewing sarcoma lines have different levels of EWSR1::FLI1 transcript. But the metric shown in Figure 2D is the percentage of cells with detectable transcript, not transcript levels. Be specific about what the data are showing here.

We agree with the need to improve clarity. In the 3rd paragraph, we changed: "we hypothesized that EwS cell lines have different levels of EWS::FLI1 transcript."

To:

"We hypothesized that EwS cell lines differ in the proportion of cells expressing high levels of the EWS::FLI1 fusion transcript."
